# Lysosomal exocytosis of HSP70 stimulates monocytic BMP6 expression in Sjögren’s syndrome

**DOI:** 10.1172/JCI152780

**Published:** 2022-03-15

**Authors:** Ying-Qian Mo, Hiroyuki Nakamura, Tsutomu Tanaka, Toshio Odani, Paola Perez, Youngmi Ji, Benjamin N. French, Thomas J.F. Pranzatelli, Drew G. Michael, Hongen Yin, Susan S. Chow, Maryam Khalaj, Sandra A. Afione, Changyu Zheng, Fabiola Reis Oliveira, Ana Carolina F. Motta, Alfredo Ribeiro-Silva, Eduardo M. Rocha, Cuong Q. Nguyen, Masayuki Noguchi, Tatsuya Atsumi, Blake M. Warner, John A. Chiorini

**Affiliations:** 1 AAV Biology Section and; 2 Salivary Disorder Unit, National Institute of Dental and Craniofacial Research, NIH, Bethesda, Maryland, USA.; 3 Department of Clinical Medicine, Ribeirão Preto Medical School,; 4 Department of Stomatology, Public Health and Forensic Dentistry, School of Dentistry of Ribeirão Preto,; 5 Department of Pathology and Legal Medicine, Ribeirão Preto Medical School, and; 6 Department of Ophthalmology, Otorhinolaryngology, Head and Neck Surgery, Ribeirão Preto Medical School, University of São Paulo, São Paulo, Brazil.; 7 Department of Pathology and Infectious Diseases, University of Florida, Gainesville, Florida, USA.; 8 Division of Cancer Biology, Institute for Genetic Medicine and; 9 Department of Rheumatology, Endocrinology and Nephrology, Faculty of Medicine, Hokkaido University, Sapporo, Japan.

**Keywords:** Autoimmunity, Autoimmune diseases, Innate immunity, Rheumatology

## Abstract

BMP6 is a central cytokine in the induction of Sjögren’s syndrome–associated (SS-associated) secretory hypofunction. However, the upstream initiation leading to the production of this cytokine in SS is unknown. In this study, RNA ISH on salivary gland sections taken from patients with SS indicated monocytic lineage cells as a cellular source of BMP6. RNA-Seq data on human salivary glands suggested that TLR4 signaling was an upstream regulator of BMP6, which was confirmed by in vitro cell assays and single-cell transcriptomics of human PBMCs. Further investigation showed that HSP70 was an endogenous natural TLR4 ligand that stimulated BMP6 expression in SS. Release of HSP70 from epithelial cells could be triggered by overexpression of lysosome-associated membrane protein 3 (LAMP3), a protein also associated with SS in several transcriptome studies. In vitro studies supported the idea that HSP70 was released as a result of lysosomal exocytosis initiated by LAMP3 expression, and reverse transcription PCR on RNA from minor salivary glands of patients with SS confirmed a positive correlation between *BMP6* and *LAMP3* expression. BMP6 expression could be experimentally induced in mice by overexpression of LAMP3, which developed an SS-like phenotype. The newly identified LAMP3/HSP70/BMP6 axis provided an etiological model for SS gland dysfunction and autoimmunity.

## Graphical abstract

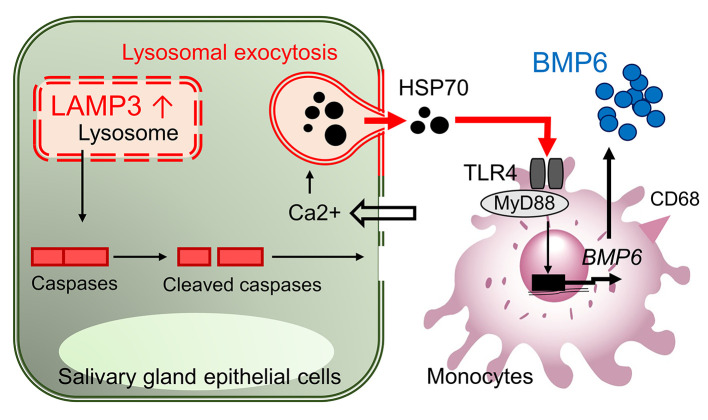

## Introduction

Sjögren’s syndrome (SS) is an autoimmune disease characterized by dry mouth and/or eye symptoms (sicca symptoms) and lymphocytic inflammation in salivary and/or lacrimal glands. The pathogenetic mechanism driving the disease has been poorly understood, although it may involve a combination of environmental and genetic factors. Because of the unknown etiology, only palliative treatment exists for SS, and many patients with SS experience a poor quality of life. A better understanding the pathophysiology is essential to develop a disease-modifying therapeutic approach for SS (1).

A complex network of cytokines and chemokines is associated with the pathogenesis of SS (2). The cytokine BMP6 belongs to the TGF-β superfamily and plays an important role in endochondral bone formation (3) and iron-related regulation of hepcidin in the liver (4). A microarray study showed increased expression of *BMP6* in the salivary glands of patients with SS compared with control salivary glands. Functional genomic studies demonstrated that BMP6 can inhibit membrane water permeability in salivary gland epithelial cells via downregulation of aquaporin 5 (AQP5) and that local overexpression of BMP6 in the salivary or lacrimal glands induces the loss of fluid secretion in mice (5). Additional studies demonstrated that inhibition of BMP6 signaling with small molecules (6) or bypassing the defect in fluid movement through aquaporin gene therapy (7) can ameliorate SS-like sicca symptoms in BMP6-overexpressing mice. These results suggest that BMP6 expression is associated with the development of secretory hypofunction in SS and is a potential therapeutic target in SS. However, the cells responsible for BMP6 expression in salivary glands have not been identified, and the upstream initiation of BMP6 expression in SS is unknown. In this study, we found that a subset of monocytic cells could express BMP6 following TLR4 stimulation and identified a signaling pathway that initiated the synthesis of BMP6 as a result of changes in lysosomal activity triggered by lysosome-associated membrane protein 3 (LAMP3) expression in salivary gland epithelial cells.

## Results

### Monocytic lineage cells express BMP6 in the salivary glands of patients with SS.

**Figure 1:**
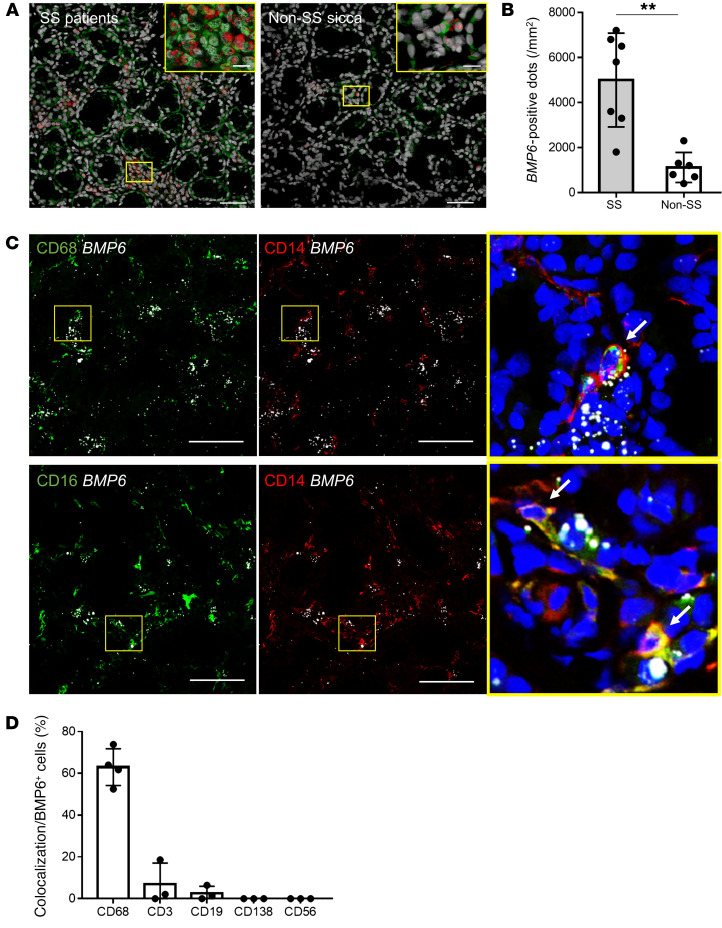
*BMP6* mRNA–expressing cells in salivary glands of patients with SS. (**A**) Representative images of dual ISH for BMP6 mRNA (red) and IF for BMP6 protein (green) and nuclei (gray) on sections of labial minor salivary glands from patients with SS or non-SS sicca. Scale bars: 50 μm and 10 μm (insets). (**B**) Mean (± SD) number of *BMP6*^+^ dots per mm^2^ (*n* = 7 patients with SS and *n* = 6 non-SS sicca). **P < 0.01, by Student’s *t* test. (**C**) Representative images of dual ISH for *BMP6* (white) and IF for CD14 (red), CD16, and CD68 (green) and nuclei (DAPI, blue) on sections of labial minor salivary glands from patients with SS. Scale bars: 50 μm. Enlargement original magnification, 100×. White arrows indicate colocalization of signals. (**D**) Percentage (mean ± SD) of colocalization with each cell marker in *BMP6*^+^ cells (*n =* 4 for CD68 and *n =* 3 for the others).

BMP6 is a secreted protein that can associate with extracellular matrix (8). Confocal immunofluorescence (IF) imaging of BMP6 protein expression indeed showed a broad staining pattern in salivary glands of patients with SS (Figure 1A). On the other hand, ISH revealed a more restricted expression pattern of *BMP6* mRNA, mainly in interstitial infiltrating cells (Figure 1A). Quantitative analysis confirmed that *BMP6* signal was significantly increased in the salivary glands of patients with SS compared with non-SS sicca individuals (Figure 1B).

To identify the cellular source of *BMP6* expression among the infiltrating cells, ISH of *BMP6* was combined with IF staining for various surface molecules of immune cells found in the salivary glands of patients with SS. The dual labeling showed that the majority of *BMP6*^+^ cells expressed monocytic lineage markers such as CD68, CD14, and CD16 (Figure 1, C and D), whereas little *BMP6* was colocalized with T cells (CD3^+^), B cells (CD19^+^), plasma cells (CD138^+^), or NK cells (CD56^+^) (Figure 1D and Supplemental Figure 1; supplemental material available online with this article; https://doi.org/10.1172/JCI152780DS1). We also detected sporadic *BMP6* expression in nonimmune cells including salivary epithelial cells, vascular endothelial cells, and fibroblasts. Taken together, these data suggested that cells of the monocytic lineage were the main source of *BMP6* expression in the salivary glands of patients with SS.

### The TLR4 pathway is associated with BMP6 expression in the salivary glands of patients with SS.

**Figure 2:**
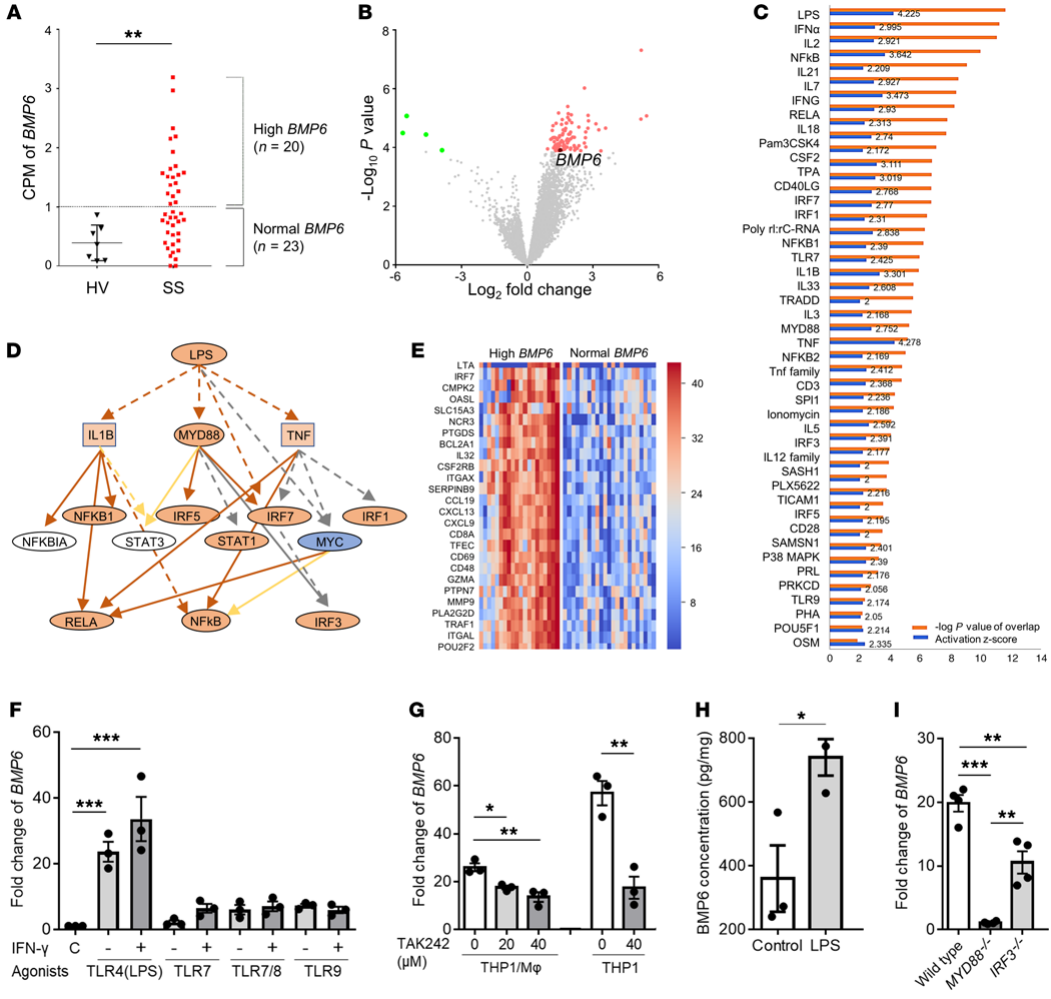
*BMP6* expression is simulated via the TLR4/MyD88-dependent pathway. (**A**) CPM of *BMP6* in RNA-Seq analysis of labial minor salivary glands from patients with SS (*n =* 43) and age-matched HVs (*n =* 7). (**B**) Volcano plot showing DEGs between patients with SS with high versus normal *BMP6* expression. (**C**) Upstream regulators of DEGs likely associated with high *BMP6* expression. (**D**) Mechanistic network for LPS signaling. (**E**) Heatmap showing the relative expression of DEGs related to LPS. (**F**) THP1 cells were simulated with PMA for 48 hours to induce differentiation into macrophages and then treated with one of the following agonists for 4 hours: TLR4 (LPS, 100 ng/mL), TLR7 (loxoribine, 1 mM), TLR7/8 (3M002, 8 μg/mL), or TLR9 (ODN2216, 5 μM), with or without IFN-γ priming (100 U/L, 8 hours). (**G**) THP1-derived macrophages and THP1 cells were pretreated with TLR4 antagonist (TAK242) at the indicated concentrations, followed by treatment with LPS for 8 and 4 hours, respectively. MΦ, macrophage. (**H**) BMP6 protein expression was quantified in THP1 cell lysates 24 hours after simulation with LPS. (**I**) WT, *MYD88^–/–^*, or *IRF3^–/–^* THP1 cells were treated with LPS for 4 hours. *BMP6* transcript expression in cells was quantified after each treatment using the ΔΔCt method relative to *ACTB* expression. Data represent the mean ± SEM of 3 independent experiments. *P < 0.05, **P < 0.01, and ***P < 0.001, by Student’s *t* test with multiple testing correction using Dunnett’s method.

The signaling pathways involved in the upregulation of BMP6 in the salivary glands of patients with SS is unknown. In order to explore pathways that may trigger BMP6 expression, we conducted RNA-Seq analysis of labial minor salivary glands from 43 female patients with SS and 7 female age-matched healthy volunteers (HVs). Patients with SS were divided into high *BMP6* (*n =* 20) and normal *BMP6* expression (*n =* 23) groups, according to the cutoff value defined as 2-fold SD from the mean counts per million reads (CPM) of *BMP6* in HVs (Figure 2A).

Analysis of differentially expressed genes (DEGs) between the 2 SS patient groups (Figure 2B and Supplemental Table 1) identified 47 potential upstream regulators associated with high *BMP6* expression (Figure 2C). LPS, a TLR4 agonist, was the most significantly associated upstream regulator (activation *z* score: 4.225). A number of other molecules mechanistically linked to the LPS pathway were also activated (Figure 2, D and E). In addition to LPS, we identified TLR7 (activation *z* score: 2.425) and TLR9 (activation *z* score: 2.056) as likely activated upstream regulators in patients with high *BMP6* expression compared with those with normal *BMP6* expression (Figure 2C).

### BMP6 expression is induced via the TLR4/MyD88 pathway.

On the basis of the finding that TLR signaling may be involved in BMP6 expression and that the monocytic lineage could be the source of *BMP6* expression as assessed by ISH, we treated THP1 cells (human monocytic cell line) with PMA to induce differentiation into macrophages (referred to as THP1-derived macrophages). These cells were then treated with LPS (a TLR4 agonist), loxoribine (a TLR7 agonist), 3M002 (a TLR7/8 agonist), or ODN2216 (a TLR9 agonist). We found a strong induction of *BMP6* expression after treatment with LPS but observed no significant induction with loxoribine, 3M002, or ODN2216, even after IFN-γ priming to induce TLR expression (Figure 2F).

We confirmed the specificity of TLR4 in *BMP6* induction by treatment with the TLR4 antagonist TAK242. THP1-derived macrophages pretreated with TAK242 showed a significant decrease in the induction of *BMP6* expression compared with control cells (Figure 2G). Similar inhibition of *BMP6* expression was observed with TAK242 in THP1 cells without PMA pretreatment (Figure 2G). Consistent with the transcriptional induction, LPS stimulation significantly increased BMP6 protein expression in THP1 cells (Figure 2H). Taken together, these data confirmed that THP1 cells expressed BMP6 upon LPS stimulation through TLR4 in vitro.

Studies have shown that LPS stimulates 2 canonical downstream pathways of TLR4: one is myeloid differentiation primary response 88 (MyD88) dependent and leads to the activation of NF-κB and MAPKs, whereas the other is TOLL/IL-1R domain–containing adaptor-inducing IFN-β (TRIF) dependent and leads to the activation of IFN regulatory factor 3 (IRF3) (9). To elucidate which pathway is involved in BMP6 expression, we stimulated *MYD88^–/–^* or *IRF3^–/–^* THP1 cells with LPS. Compared with the parent THP1 cells, we found that LPS-induced *BMP6* expression was significantly reduced in *MYD88^–/–^* THP1 cells. In contrast, LPS induction of *BMP6* expression in *IRF3^–/–^* THP1 cells showed less inhibition (Figure 2I). These data suggested that BMP6 expression was MyD88 pathway dependent.

### HSP70 stimulates BMP6 expression via the TLR4/MyD88 pathway.

**Figure 3:**
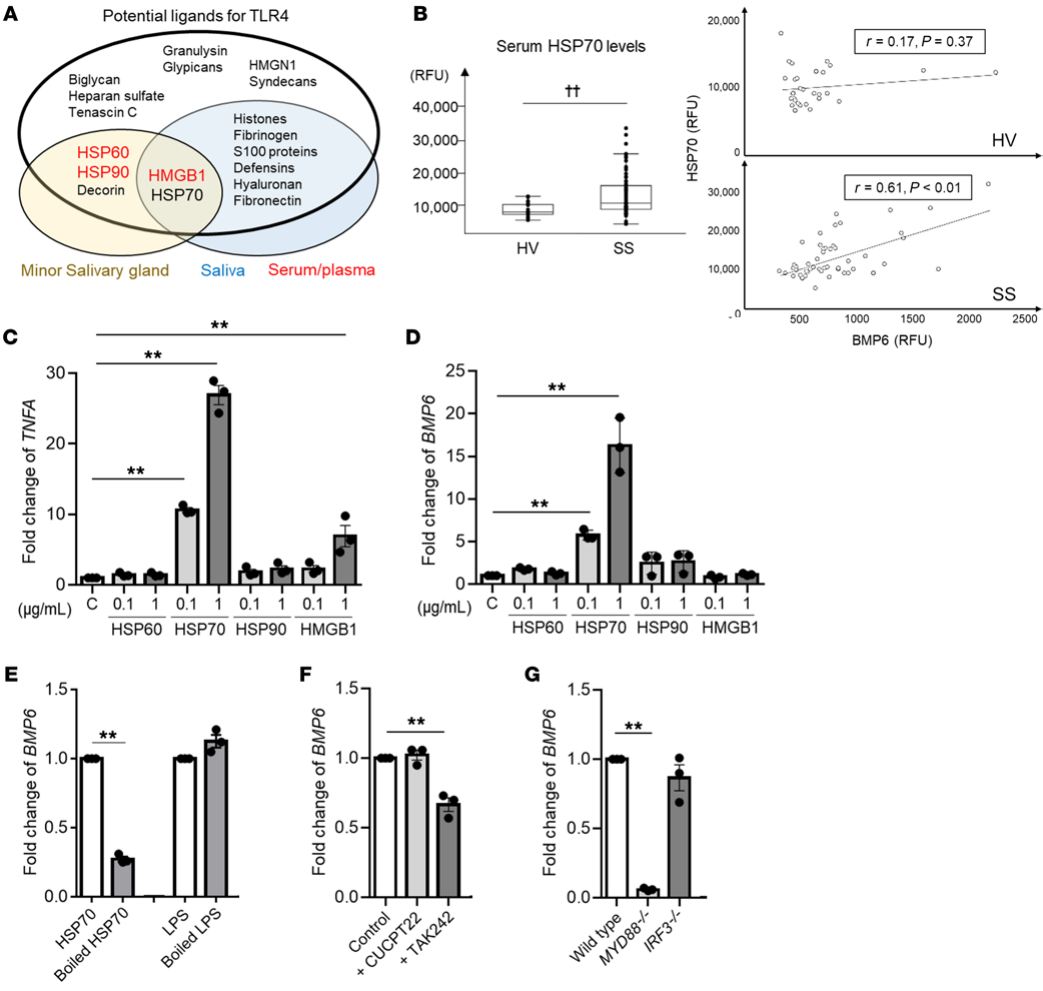
HSP70 stimulates *BMP6* expression via the TLR4/MyD88 pathway. (**A**) Venn diagram showing increased expression of potential ligands for TLR4 (listed inside the largest oval) in minor salivary glands (yellow oval), saliva (blue oval), and/or serum/plasma (shown in red) from patients with SS. (**B**) Serum HSP70 and BMP6 levels in patients with SS (*n =* 50) and HVs (*n =* 30). Boxes represent first, second, and third quartiles. ^††^*P <* 0.01, by Wilcoxon test. RFU, relative fluorescence units. (**C** and **D**) THP1 cells were treated with the indicated recombinant proteins at the indicated concentration for 4 hours. C, control. (**E**) THP1 cells were treated with boiled HSP70 (1 μg/mL) or LPS (100 ng/mL) for 4 hours. (**F**) THP1 cells were pretreated with TLR1/2 antagonist (CUCPT22, 20 μM) or TAK242 (40 μM) 1 hour prior to HSP70 simulation. (**G**) WT, *MYD88^–/–^*, or *IRF3^–/–^* THP1 cells were stimulated with HSP70 for 4 hours. *TNFA* and *BMP6* transcript levels in the cells were quantified after each treatment using the ΔΔCt method relative to *ACTB* expression. Values shown are mean ± SEM of 3 independent experiments. **P < 0.01, by Student’s *t* test with multiple testing correction using Dunnett’s method (**C**–**G**).

The previous data demonstrated that TLR4 activation by LPS can induce BMP6 expression in vitro; however, it is unclear whether LPS functions as a natural ligand for BMP6 expression in patients with SS. Certain endogenous ligands, called damage-associated molecular patterns (DAMPs), are able to trigger a similar response through TLR4 (10). Following a systematic literature search and review of publicly available proteomics data sets (Supplemental Table 2), we identified several candidates as potential SS-associated TLR4 ligands. HSP60, HSP70, HSP90, high-mobility group box 1 (HMGB1), and decorin are all increased in minor salivary glands of patients with SS (11, 12). Furthermore, DAMPs including HSP70, HMGB1, S100, and histones are increased in the saliva of patients with SS (13–15). Increased serum/plasma HSP60, HSP90, and HMGB1 levels have also been reported in patients with SS (refs. 16–19, Figure 3A, and Supplemental Table 3). Our SomaScan data showed significantly higher serum HSP70 levels in an independent cohort of patients with SS compared with HVs (Figure 3B). Analysis of the serum HSP70 levels revealed a significant correlation with serum BMP6 levels in patients with SS (*r* = 0.61, *P <* 0.01), but not in HVs (*r* = 0.17, *P =* 0.37; Figure 3B). As HSP60, HSP70, HSP90, and HMGB1 are common enriched proteins in salivary glands, saliva, and serum/plasma, we further studied them as candidate ligands for inducing BMP6 expression in SS.

Among the potential TLR4 ligands, treatment of THP1 cells with recombinant HSP70 or HMGB1 protein stimulated a significant increase in the transcription of *TNFA,* which is induced by downstream signaling of TLR4, while recombinant HSP60 and HSP90 did not (Figure 3C). Treatment with HSP70 significantly increased the transcription of *BMP6* compared with control THP1 cells, whereas treatment with HSP60, HSP90, or HMGB1 did not result in increased *BMP6* expression (Figure 3D). As a control for potential LPS contamination in the recombinant HSP70 protein, the protein was denatured by boiling at 100°C for 1 hour (20). Boiled HSP70 failed to induce *BMP6* expression in THP1 cells (Figure 3E).

To determine whether HSP70 stimulates *BMP6* expression through TLR4, we pretreated THP1 cells with TAK242 (a TLR4 antagonist) or CUCPT22 (TLR1/2 antagonist) 1 hour before HSP70 stimulation. THP1 cells pretreated with TAK242 showed a significant decrease in the induction of *BMP6,* whereas pretreatment with CUCPT22 did not alter *BMP6* expression significantly (Figure 3F). Similar to LPS induction, HSP70 induction of *BMP6* was significantly reduced in *MYD88^–/–^* THP1 cells but unaffected in *IRF3^–/–^* THP1 cells (Figure 3G). Again, our data supported the notion that *BMP6* expression induced by a TLR4 agonist is dependent on the MyD88 pathway.

### BMP6 expression is upregulated via the TLR4 pathway in human monocytes.

To confirm whether BMP6 expression is regulated through the same pathway in primary cells and to identify the BMP6-expressing subpopulation of cells, single-cell RNA-Seq libraries were generated from human PBMCs stimulated with a sham control, LPS, or HSP70. To enrich for monocytes, cells were allowed to separate into adherent and nonadherent subgroups during in vitro culturing and were then profiled using the 10× Genomics Chromium Droplet platform (21). Following quality control to remove low-quality cells and doublets, our analysis showed that half of the adherent PBMCs were monocytes, whereas T cells were dominant in the nonadherent PBMCs (Supplemental Figure 2, A and B). The activation state of TLR4 in PBMCs treated with LPS or HSP70 was confirmed by upregulation of *TNFA*, *IL6*, and *IL10* (Supplemental Figure 2C).

**Figure 4:**
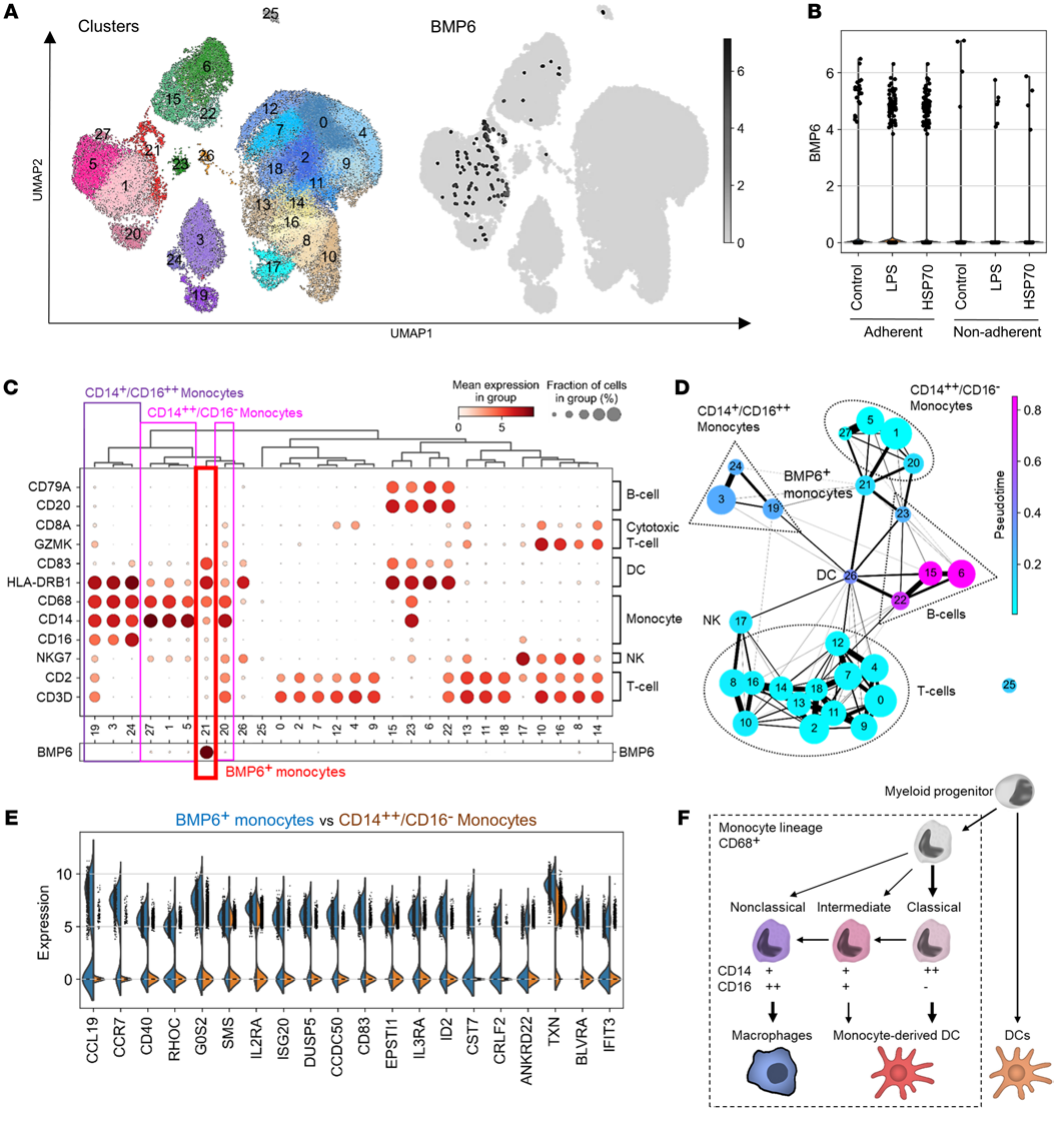
*BMP6* expression is upregulated via the TLR4 pathway in human monocytes. Human PBMCs were treated with sham control, LPS (100 ng/mL), or HSP70 (1 μg/mL) for 20 hours and then analyzed using the 10× Genomics platform. (**A**) Clustered PBMCs are displayed in UMAP format with the distribution of cells expressing *BMP6*. (**B**) Relative expression of *BMP6* and the number of *BMP6^+^* cells in each group. (**C**) Proportion and relative expression of the indicated genes in each cell cluster. (**D**) Connectivity and trajectory among cell clusters. (**E**) Top 20 DEGs between *BMP6*^+^ monocytes and *CD14*^++^*CD16^–^* (classical) monocytes. (**F**) General differentiation pathway of the monocyte lineage.

Single-cell RNA-Seq analysis of the PBMCs identified 28 unique cell clusters (Figure 4A). *BMP6*-expressing cells were mainly present in the adherent cell population, and the number of *BMP6*^+^ cells was amplified by treatment with LPS or HSP70 compared with sham-treated cells (Figure 4B). Various *CD68*^+^ monocytic clusters were identified in the PBMCs. Clusters 1, 5, 20, and 27 were *CD68*^+^ and *CD14*^+^ but *CD16*^–^, which is consistent with the gene expression profile of classical monocytes. In addition, clusters 3, 19, and 24 were *CD68*^+^, *CD14*^+^, and *CD16*^+^, which corresponded with nonclassical monocytes. Cluster 21 was also *CD68*^+^ and thus considered as part of the monocytic lineage. The majority of *BMP6*-expressing cells belonged to cluster 21 (Figure 4C), confirming that monocytic lineage cells are the predominant cell population expressing *BMP6* among PBMCs.

The *BMP6^+^* monocyte cluster showed lower expression of *CD14* and *CD16* than did the other classical and nonclassical monocyte clusters, but higher expression of *HLA-DRB1* and *CD83* (Figure 4C). Further analysis of the single-cell transcriptomic data set using the pseudotime axis suggested connectivity among the *BMP6^+^* monocytes, classical (*CD14*^++^*CD16*^−^) monocytes, and DCs and a trajectory from classical monocytes to DCs via the *BMP6^+^* monocytes (Figure 4D). A comparison between *BMP6^+^* monocytes and classical monocytes revealed an upregulation of mature DC–associated genes, such as *CCL19*, *CCR7*, *CD40*, *DUSP5*, *CCDC50*, *CD83*, and *IL3RA*, in the *BMP6*^+^ monocytes (Figure 4E). In reference to the general differentiation pathway of the monocytic lineage (refs. 22–24 and Figure 4F), *BMP6^+^* monocytes could be considered a subtype of classical monocytes with a gene expression profile associated with monocyte-derived DCs.

Consistent with the single-cell transcriptomic data, LPS stimulation significantly induced *BMP6* expression in PBMCs, especially in the adherent cell population (Supplemental Figure 3A). We observed no significant difference in LPS stimulation of *BMP6* between the SS and HV samples (Supplemental Figure 3A). TAK242 significantly inhibited LPS induction of *BMP6* in the adherent PBMCs (Supplemental Figure 3B). HSP70 treatment significantly increases *BMP6* transcript expression in PBMCs, particularly in the adherent cell population (Supplemental Figure 3C). This increase was impaired by TAK242 but not by CUCPT22, confirming a TLR4-dependent induction of BMP6 in primary cells.

### LAMP3 expression in salivary gland epithelial cells contributes to BMP6 expression via the release of HSP70.

**Figure 5:**
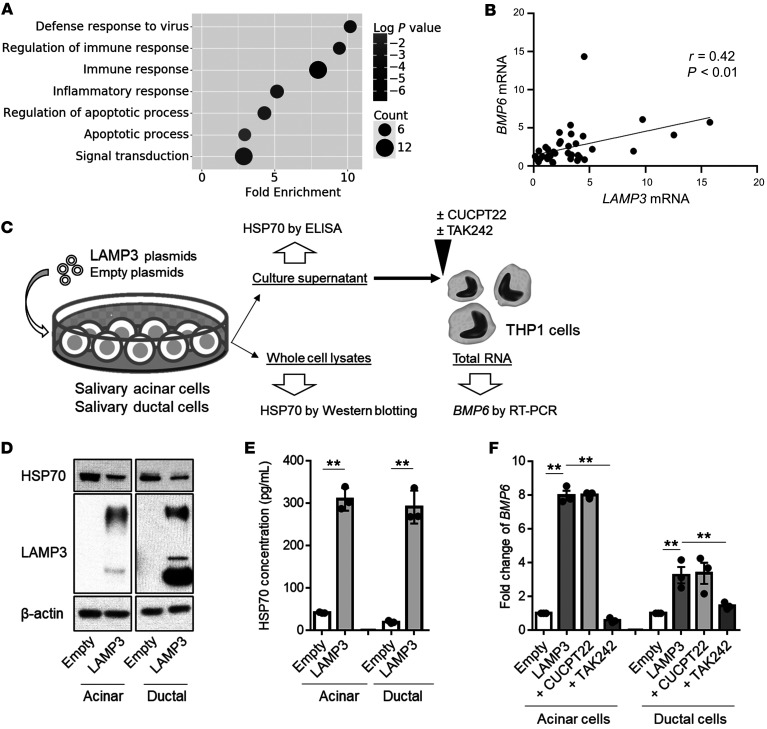
LAMP3 expression in salivary gland epithelial cells is involved in *BMP6* expression by the release of HSP70. (**A**) Scatter plot showing enriched gene ontology in patients with SS with high *BMP6* expression compared with those with normal *BMP6* expression. (**B**) Correlation between *LAMP3* and *BMP6* transcript expression in minor salivary glands of patients with SS (*n =* 43). (**C**) Schematic showing the methods used in the in vitro assays. (**D**) Representative Western blot with the indicated antibodies using lysates of salivary acinar or ductal cells 72 hours after transfection with empty and/or *LAMP3* expression plasmids. (**E**) HSP70 concentration in culture supernatant collected 96 hours after transfection. (**F**) THP1 cells were treated with the culture supernatant of acinar or ductal cells with or without CUCPT22 (20 μM) or TAK242 (40 μM). *BMP6* transcript levels in THP1 cells were evaluated 20 hours after stimulation using the ΔΔCt method relative to *ACTB*. Values shown are the mean ± SEM of 3 independent experiments. **P < 0.01, by Student’s *t* test with multiple testing correction using Tukey’s method (**E** and **F**).

The previous results revealed that HSP70 was able to stimulate *BMP6* expression via TLR4. However, it is unknown how extracellular HSP70 levels are increased in patients with SS. Gene ontology analysis of the RNA-Seq data indicated the upregulation of genes related to apoptosis and inflammatory and immune processes in patients with SS who had high expression of *BMP6* (Figure 5A). Recent studies showed that LAMP3 is overexpressed in patients with SS, which increases apoptosis and the release of DAMPs in salivary gland epithelial cells, leading to immune cell activation via TLRs (25). Our analysis demonstrated that *LAMP3* expression was significantly correlated with *BMP6* expression (*r* = 0.42, *P <* 0.01) in minor salivary glands of patients with SS (Figure 5B), indicating an association between LAMP3 and BMP6 in SS.

Although it is possible that LAMP3 directly stimulates the expression of BMP6 in monocytes, the increased BMP6 expression may also be an indirect result of released DAMPs following the overexpression of LAMP3 in epithelial cells. To test if LAMP3 expression in epithelial cells is involved in extracellular release of HSP70, we measured the HSP70 concentration in culture supernatant of salivary acinar cells (NS-SV-ACs) and salivary ductal cells following the overexpression of LAMP3 (Figure 5C). Although Western blotting showed that LAMP3 did not increase HSP70 expression in the cells (Figure 5D), LAMP3 expression significantly increased the HSP70 concentration in culture supernatants of the transfected cells (Figure 5E).

Consistent with this result, treatment of THP1 cells with supernatant from LAMP3-overexpressing epithelial cells led to a significant increase in the transcription of *BMP6* compared with that seen in control cells. We found that the increased expression of *BMP6* in THP1 cells was inhibited by pretreatment with TAK242 but not CUCPT22 (Figure 5F). These data demonstrated that LAMP3 expression in salivary gland epithelial cells could stimulate *BMP6* expression by the release of HSP70.

### LAMP3 promotes the release of HSP70 by caspase-dependent lysosomal exocytosis.

The previously described data suggested that HSP70 can be released from salivary gland epithelial cells following alterations in lysosomal function initiated by LAMP3 expression. A previous study showed that LAMP3 expression promotes the release of some proteins via extracellular vesicles (EVs) (26). To determine whether HSP70 is associated with EVs, we treated THP1 cells with isolated EVs from the supernatant of LAMP3-overexpressing epithelial cells (Supplemental Figure 4A). Although significant *BMP6* expression was stimulated by treatment with unfractionated supernatant, treatment with EVs alone did not increase *BMP6* expression in THP1 cells (Supplemental Figure 4, B–D). Furthermore, *BMP6* induction with supernatant from LAMP3-overexpressing epithelial cells was blocked by pretreatment with an HSP70-neutralizing antibody (Supplemental Figure 4E), suggesting that free HSP70 in the supernatant could stimulate *BMP6* expression.

**Figure 6:**
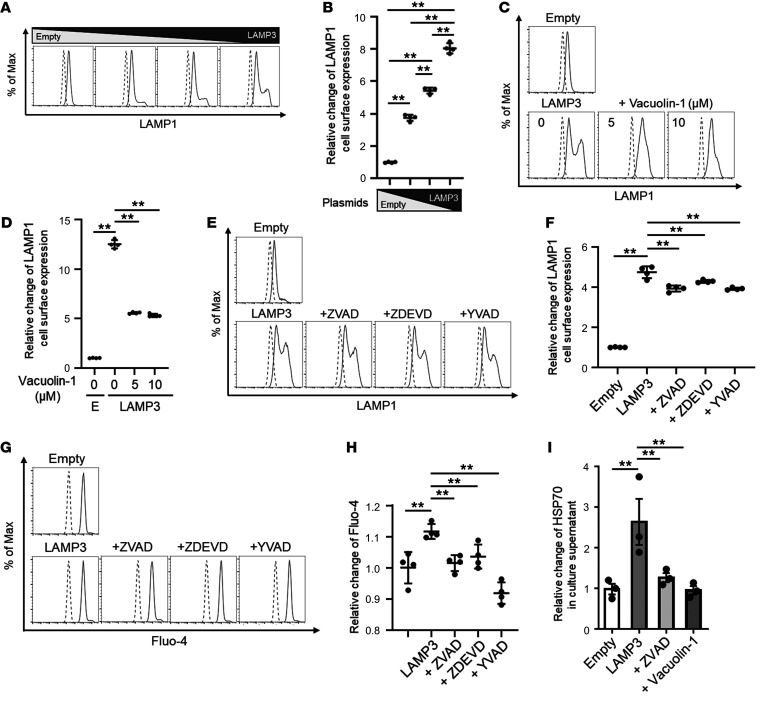
HSP70 is released by caspase-dependent lysosomal exocytosis from LAMP3-overexpressing epithelial cells. (**A**–**H**) HSG cells were transfected with empty and/or *LAMP3* expression plasmids and then treated with vacuolin-1 (at the indicated concentration), ZVAD (20 μM), ZDEVD (10 μM), or YVAD (50 μM). Lysosomal exocytosis was monitored by quantifying LAMP1 expression on the cell surface, and the intracellular Ca^2+^ concentration was determined by Fluo-4 fluorescence by flow cytometry 48 hours after transfection. (**I**) The HSP70 concentration was evaluated in culture supernatant collected from HSG cells 72 hours after transfection with or without ZVAD (20 μM) or vacuolin-1 (10 μM). Values shown are the mean ± SEM from 3 (**I**) or 4 (**A**–**H**) independent experiments. **P < 0.01, by Student’s *t* test with multiple testing correction using Tukey’s method (**B**) or Dunnett’s method (**D**, **F**, **H**, and **I**). Max, maximum.

A main pathway involved in the release of HSP70 is lysosomal exocytosis (27). This process leads to the translocation of LAMP1 to the plasma membrane and secretion of lysosomal content upon lysosome fusion with the plasma membrane. Lysosomal exocytosis plays a physiological role in intercellular signaling and plasma membrane repair (28). To test if LAMP3 expression promotes lysosomal exocytosis, we performed flow cytometry to assess cell surface trafficking of LAMP1 in LAMP3-overexpressing epithelial cells (29). We found that LAMP3 expression significantly increased LAMP1 expression on the cell surface (Figure 6, A and B). The redistribution of LAMP1 protein to the plasma membrane by LAMP3 was inhibited by vacuolin-1 (Figure 6, C and D), an inhibitor of lysosomal exocytosis that prevents Ca^2+^-dependent fusion of lysosomes with the plasma membrane (30), and by a pan-caspase inhibitor (ZVAD), a caspase-3 inhibitor (ZDEVD), or a caspase-1 inhibitor (YVAD) (Figure 6, E and F). These results demonstrated that LAMP3 expression could induce caspase-dependent lysosomal exocytosis of cellular proteins.

Consistent with the presence of this mechanism, LAMP3 expression increased the intracellular Ca^2+^ concentration as monitored by Fluo-4 fluorescence, and this increase was inhibited by the same caspase inhibitors (Figure 6, G and H). Treatment with vacuolin-1 or ZVAD significantly decreased extracellular HSP70 levels in LAMP3-overexpressing epithelial cells (Figure 6I). These results indicated that LAMP3 promoted the release of HSP70 through caspase-dependent lysosomal exocytosis.

### LAMP3 expression stimulates BMP6 expression via TLR4 in vivo.

**Figure 7:**
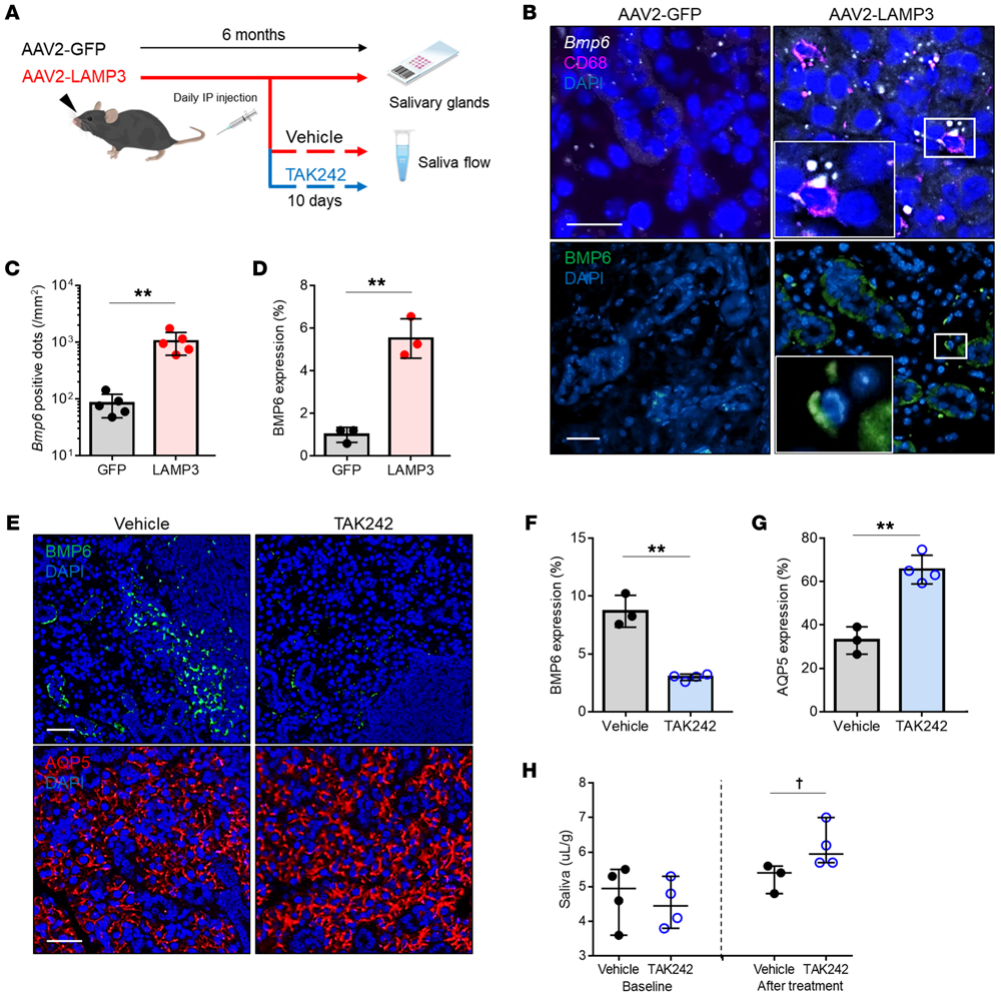
Epithelial LAMP3 expression stimulates monocytic BMP6 expression via TLR4 in murine salivary glands. (**A**) Submandibular glands of C57BL/6 mice were instilled with an AAV2 vectors encoding LAMP3 (AAV2-LAMP3) or GFP (AAV2-GFP), and the tissues and saliva flow rate were evaluated 6 months later. Mice were given i.p. injections of TAK242 or vehicle for the last 10 days. (**B**) Representative images of dual ISH for *Bmp6* (white) and IF for CD68 (magenta) and nucleus (DAPI, blue) (upper panel), and images of IF for BMP6 (green) and nuclei (DAPI, blue) (lower panel) in submandibular gland sections. Scale bars: 20 μm. Enlargement original magnification, 100×. Quantification of (**C**) *Bmp6^+^* dots per mm^2^ (*n =* 5 each) and (**D**) BMP6 expression area (*n =* 3 each). (**E**) Representative IF images for BMP6 (green, upper panel), AQP5 (red, lower panel), and nuclei (DAPI, blue) in submandibular gland sections. Scale bars: 20 μm. Quantification of (**F**) BMP6 and (**G**) AQP5 expression area. Values shown are the mean ± SD. **P < 0.01, by Student’s *t* test (**C**, **D**, **F**, and **G**). (**H**) Pilocarpine-stimulated salivary flow per body weight over 20 minutes in TAK242-treated mice (*n =* 4) and vehicle-treated mice (*n =* 3). Values shown indicate the median and the range. ^†^*P <* 0.05, by Wilcoxon test.

The previous in vitro studies suggested that LAMP3 expression promoted the release of HSP70 by lysosomal exocytosis, which stimulated BMP6 expression via TLR4. To test the causal relationship in vivo, we induced LAMP3 expression in the salivary glands of healthy C57BL/6 mice by retroductal cannulation of the submandibular glands using adeno-associated virus serotype 2 (AAV2) vectors encoding LAMP3 (AAV2-LAMP3) or GFP (AAV2-GFP) (Figure 7A). AAV2-LAMP3–treated mice developed an SS-like phenotype with progressive salivary hypofunction and autoantibody production as previously reported (25).

We evaluated BMP6 expression in murine submandibular glands by ISH and/or IF 6 months after cannulation. The ISH study showed induction of *Bmp6* mRNA expression in submandibular glands from AAV2-LAMP3–treated mice compared with those from control AAV2-GFP–treated mice. Dual labeling demonstrated that *Bmp6*-expressing cells were positive for CD68 (Figure 7B). Confocal IF imaging showed BMP6 protein in the interstitial infiltrating cells and a broad distribution of the secreted protein in the submandibular gland specimens taken from AAV2-LAMP3–treated mice (Figure 7B). The increased *Bmp6* mRNA and BMP6 protein expression in the submandibular glands of AAV2-LAMP3–treated mice supports a causal relationship between LAMP3 and BMP6 expression in vivo (Figure 7, C and D). To confirm the role of TLR4 in the induction of BMP6 following stimulation, AAV2-LAMP3–treated mice with established salivary hypofunction were treated i.p. with the TLR4 antagonist TAK242 for 10 days, and the effect of the drug on salivary gland protein expression and function was tested. We observed that treatment with TAK242 significantly decreased BMP6 expression (Figure 7, E and F) and increased AQP5 expression (Figure 7, E and G) in the salivary gland tissues. In agreement with this observation, the saliva flow rate of AAV2-LAMP3–treated mice also increased compared with that in vehicle control–treated mice (Figure 7H).

Additionally, we assessed the relationship among LAMP3, TLR4, and BMP6 in human salivary glands using IF to quantify expression levels. Analysis of the expression of these proteins showed an increase in salivary glands of patients with SS compared with those of non-SS sicca individuals (Supplemental Figure 5, A and B). Furthermore, BMP6 expression showed a significant correlation with LAMP3 (*r* = 0.89, *P <* 0.01) and TLR4 (*r* = 0.94, *P <* 0.01) expression (Figure 5C), again supporting an association among LAMP3, TLR4, and BMP6 in SS.

## Discussion

SS is often referred to as an epithelitis, but a systematic etiology of SS has not been established because of the heterogeneity of the disease. Increased expression of LAMP3 (26) and BMP6 (5) and their contribution to the pathophysiology of SS have been reported in independent studies, but no pathological link between the 2 proteins was evident. LAMP3 expression is regulated by transcription factor 4 in response to endoplasmic reticulum stress (31) and is reported to be induced by type I IFN (32), whereas the mechanisms involved in the transcriptional regulation of BMP6 are yet unknown. In this study, we showed that LPS induced BMP6 expression via a TLR4/MyD88-dependent pathway in monocytes. In addition, we demonstrated that extracellular HSP70 could act as a natural TLR4 ligand and could stimulate BMP6 expression in vitro. Further analysis revealed that HSP70 was released from LAMP3-overexpressing salivary gland epithelial cells through caspase-dependent lysosomal exocytosis. These experimental data were supported by clinical data on patients with SS, who had increased extracellular HSP70 levels in saliva and serum and a positive correlation among LAMP3, TLR4, and BMP6 expression in their salivary glands. In addition, our mouse experiments confirmed the causal relationship between the 2 proteins in salivary glands. Activation of the LAMP3/BMP6 pathway via lysosomal exocytosis of HSP70 appeared to be unique to the pathogenesis of SS. Both of their expression levels were specifically upregulated in the salivary glands of patients with SS compared with those of healthy or non-SS sicca individuals, including in IgG4-related disease as shown in this study and a previous publication (26).

SS is associated with an inflammatory response in the secretory epithelium, which is likely driven by microbial or viral infection (33–35). Several different types of infections increase the risk of SS by activating the innate immune system, creating proinflammatory microenvironments and promoting immune-mediated sialadenitis (36). LAMP3 is an IFN-inducible gene, and viral infection can cause LAMP3 expression in salivary glands as the first step in the initiation of SS (37, 38). Here, we describe that this step is followed by BMP6 expression stimulated by extracellular HSP70, which is released from LAMP3-overexpressing salivary gland epithelial cells. Considering that BMP6 expression was similarly induced by TLR4 stimulation in monocytes from HVs as well as in those from patients with SS, a critical step in the LAMP3/HSP70/BMP6 axis we identified is the expression of LAMP3 in the release of HSP70 via lysosomal exocytosis. Recent work showed that LAMP3 expression alters lysosome function and activates a cascade of caspase-dependent apoptotic pathways (26). This study showed that LAMP3-mediated lysosomal exocytosis is also caspase dependent and overlaps with the apoptotic pathway. Our results highlight a critical role for lysosomal trafficking in the pathophysiology of SS. Our data suggest that stabilization of lysosomes may be a promising therapeutic approach for the treatment of SS.

Previous studies showed a significant increase in monocyte lineage cells, as well as high levels of the related cytokines in salivary glands preceding the arrival of T and B lymphocytes, suggesting that monocytes are potent cytokine secretors under inflammatory circumstances in the development of SS (39). However, an innate immune event mediated by monocytes has been poorly characterized in spite of extensive research on an abnormal adaptive immune response associated with T and B lymphocytes in SS. Recent studies showed that LAMP3 expression in salivary gland epithelial cells is associated with immune cell activation by increased apoptosis and autoantigen release (25, 26), and that extracellular HSP70 possesses several immunomodulatory functions distinct from its intracellular role as a chaperone (40). In addition, we demonstrated that HSP70 could stimulate BMP6 expression in monocytes. BMP6 contributes to further immune activation by enhancing T cell proliferation and Th1/Th17 differentiation in mesenchymal stem cells (41, 42). Our single-cell transcriptomic analysis identified the unique subset of monocytes expressing BMP6. The BMP6^+^ monocytes in PBMCs were *CD16^+/−^*, but differentiated into *CD16*^+^ cells following infiltration into the salivary glands (22). This subset had a gene expression profile showing a linage trajectory toward monocyte-derived DCs rather than macrophages. Monocyte-derived DCs are a distinct DC subset involved in inflammatory and autoimmune responses (43). It has been suggested that monocyte-derived DCs are associated with SS pathophysiology through their activity as antigen-presenting and cytokine-producing cells (44). The majority of monocyte-derived DCs are *CD68^+^CD14^+^CD16^+^* (22), consistent with the staining pattern we observed. Overall, our work highlights a critical role of monocytes in the initiation of autoimmunity in SS.

TLRs are essential components of the innate immune response. Among the large family of TLRs, TLR4 expression is significantly increased in minor salivary glands of patients with SS compared with those of controls, with intense staining reported on infiltrating mononuclear cells (45). Consistent with this finding, we detected BMP6 expression in monocytic lineage cells in the salivary glands of patients with SS. Interestingly, knockout of *MYD88* prevented the development of symptoms in a mouse model of SS, suggesting that MyD88 is required for the development of an SS-like phenotype (46). These findings suggest that TLR4 and MyD88 are probably activated and initiate SS via the induction of innate immune response.

Our identification of the LAMP3/HSP70/BMP6 axis connects immune stimulation with changes in epithelial cell function. In addition to BMP6, other proinflammatory cytokines are likely to be triggered by the same release of DAMPs following LAMP3-mediated lysosomal exocytosis. In the future, the pathophysiological role of DAMPs in SS should be comprehensively studied in relation to BMP6 and LAMP3. Additional work demonstrated that BMP6 expression is able to influence the secretory epithelial cells of the salivary gland and block their function (5). Further research is required to understand whether BMP6 expression is part of a positive feedback loop and can stimulate LAMP3 expression and perpetuate the proinflammatory state associated with SS. Other studies with small-molecule inhibitors of BMP6 signaling have suggested that by blocking the effect of BMP6, the loss of gland function and inflammation can be reversed in SS-like animal models with established disease (6).

SS has been described as an autoimmune epithelitis (1). A critical finding in this study was the interplay and communication between cell populations within the salivary glands and the central role that the epithelia played in stimulating the immune response. By identifying the cell population and a signaling pathway responsible for the LAMP3/HSP70/BMP6 axis of connection, other therapeutic molecules that block lysosomal exocytosis, TLR4 activation, and/or induction of monocytes may be identified that can reverse the development of SS.

## Methods

### Clinical samples.

Labial minor salivary gland biopsies were obtained from female patients with SS who fulfilled the 2002 American-European Consensus Group criteria, from non-SS sicca individuals who had sicca symptoms but did not meet the classification criteria for SS, and from age-matched HVs recruited at the Sjögren’s Syndrome Clinic of the NIDCR, the Clinical Hospital of the Medical School of Ribeirão Preto of the University of São Paulo, and the Hokkaido University Hospital. Serum samples and PBMCs were obtained from the NIDCR Sjögren’s Syndrome Clinic at the NIDCR. The patients’ characteristics are shown in Supplemental Table 4.

### Animals.

Female 6- to 8-week-old C57BL/6 mice were obtained from Charles River Laboratories. AAV2 vectors encoding LAMP3 or GFP were delivered to both submandibular glands (10^11^ particles/mouse in 100 μL PBS) by retrograde ductal instillation through a thin cannula as described previously (25). Mice received i.p. TAK242 (MedChemExpress) at 100 μg/BW/d (3–4 mg/kg) in a vehicle of 1% DMSO (MilliporeSigma) diluted in PBS for 10 days.

### Cells.

THP1 cells were purchased from American Type Culture Collection (ATCC) and grown in RPMI-1640 culture medium (Thermo Fisher Scientific) supplemented with 10% FBS, 2 mM l-glutamine, and 0.05 mM 2-ME. THP1 cells were treated with 100 nM PMA (MilliporeSigma) in 2-ME–free RPMI-1640 medium with 10% FBS to induce differentiation into macrophages, and 48 hours later, the medium was replaced with PMA-free medium. *MYD88^–/–^* or *IRF3^–/–^* THP1 cells were purchased from InvivoGen and grown in RPMI-1640 culture medium supplemented with 10% FBS, 2 mM l-glutamine, 25 mM HEPES, and 2 selection antibiotics. These cell lines were further engineered to produce secreted embryonic alkaline phosphatase (SEAP) and Lucia luciferase as indicators of the MyD88 and IRF3 pathway, respectively. The impairment of either pathway in *MYD88^–/–^* and *IRF3^–/–^* THP1 cells was verified prior to the study. SEAP production was slightly impaired in *IRF3^–/–^* THP1 cells (data from the manufacturer) because of an interaction between the 2 pathways (47).

Immortalized acinar cells (NS-SV-ACs) and ductal cells, which had been derived from normal human salivary glands, were donated by M. Azuma (Tokushima University School of Dentistry, Tokushima, Japan). These cell clones retain characteristics of salivary acinar and ductal cells, respectively (48). Both cell clones were treated with MycoZap (Lonza) and then cultured in defined keratinocyte serum-free medium (Thermo Fisher Scientific) and keratinocyte growth medium-2 (Lonza), respectively. Human submandibular gland (HSG) cells, provided by I. Ambudkar (National Institute of Dental and Craniofacial Research, NIH, Bethesda, Maryland, USA), were cultured in DMEM (Thermo Fisher Scientific) supplemented with 10% FBS.

Fresh whole blood was collected in a BD Vacutainer CPT, and PBMCs were isolated according to the manufacturer’s instructions. PBMCs were cultured in RPMI-1640 medium supplemented with 10% FBS, 2 mM l-glutamine, and 0.05 mM 2-ME.

All cells were incubated at 37°C with humidity in 5% CO_2_.

### Reagents and plasmids.

The following chemical reagents and recombinant proteins were purchased: LPS, ZDEVD, YVAD, and vacuolin-1 (MilliporeSigma); loxoribine, 3M002, and ODN2216 (InvivoGen); ZVAD and HSP60, -70, and -90 (Enzo Life Sciences); CUCPT22 and TAK242 (Tocris Bioscience); IFN-γ and HMGB1 (R&D Systems); and mouse HSP70-neutralizing antibody (Thermo Fisher Scientific, MA3-009). Recombinant HSP70 protein was denatured by boiling at 100°C for 1 hour (20) as a control to detect potential LPS contamination.

Expression plasmids pME18S-empty and pME18S-LAMP3 were prepared as described previously (26). Acinar and ductal cells were transfected with a total amount of 1.0 μg plasmids per 1 × 10^6^ cells using Amaxa Nucleofector (Lonza). HSG cells were transfected with a total amount of 3.0 μg plasmids per 1 × 10^6^ cells using Lipofectamine 3000 (Thermo Fisher Scientific).

### ISH and IF staining.

Salivary glands were fixed with 10% neutral buffered formalin (NBF), embedded in paraffin, and sectioned at 5 μm. Sections were assayed using a *BMP6* (no. 474451) or *Bmp6* (no. 488801) probe based on an RNAscope assay (Advanced Cell Diagnostics) according to the manufacturer’s instructions. Briefly, after deparaffinization, slides were incubated in hydrogen peroxide at room temperature for 10 minutes, in antigen retrieval reagent at 99°C for 15 minutes, and in then Protease Plus (Advanced Cell Diagnostics) at 40°C for 15 minutes. Probes were then added for 2 hours at 40°C. Slides were sequentially incubated in signal amplification and detection reagents. After the ISH protocol, IF staining was conducted on the same slides (dual ISH/IF). Colocalized signals between *BMP6* and each cell marker protein were counted on the slides, and the proportion per whole *BMP6*^+^ cells was calculated.

For IF, slides were blocked with 2% BSA (MilliporeSigma) at 25°C for 1 hour and then incubated at 4°C overnight with a primary antibody solution consisting of mouse anti-BMP6 (Abcam, ab15640); mouse anti-TLR4 (Abcam, ab22048); mouse anti-CD14 (Abcam, ab182032); rabbit anti-CD16 (Abcam, ab203883); rabbit anti-CD68 (Abcam, ab213363 and ab125212); rabbit anti-CD19 (Abcam, ab227019); mouse anti-CD56 (Abcam, ab200698); rabbit anti-CD138 (Abcam, ab128936); rat anti-CD3 (LifeSpan BioSciences, LS-B8765-50); rabbit anti-LAMP3 (Proteintech, 12632-1-AP); and/or rabbit anti-AQP5 (Alomone Labs, AQP-005) antibodies. Next, slides were washed and then incubated with Alexa Fluor 488–, 596–, or 647–conjugated secondary antibodies (Jackson ImmunoResearch) at 25°C in the dark for 1 hour, followed by washing and counterstaining with DAPI mounting medium (Abcam). All images were acquired by using a Nikon fluorescence microscope. Gene and protein expression was quantified using ImageJ software (NIH).

### Bulk RNA-Seq analysis.

Labial minor salivary gland tissues were disrupted in RLT buffer (QIAGEN) using the TissueRuptor II and disposable, single-use rotor-stators (QIAGEN). Total RNA was extracted with the RNeasy Mini Kit (QIAGEN) and treated with Turbo DNase (Thermo Fisher Scientific). RNA quality was measured using a 2100 Bioanalyzer (Agilent Technologies), and only RNA samples with a 28S/18S ribosomal RNA ratio greater than 1.7 and an RNA integrity number above 6.5 were used. cDNA libraries were produced using the Nextera XT method and then run on a NextSeq500 Sequencing System (Illumina) configured for 37 × 37 paired-end reads. For read counting, the quantMode utility of STAR aligner software was used, and normalized counts were generated per gene as CPM.

The EdgeR package was used to assess DEGs between groups (FDR < 0.05). Ingenuity Pathway Analysis (IPA) bioinformatics core analysis (QIAGEN) was used to identify molecular activity and upstream regulators of DEGs. The IPA upstream regulator module uses causal analytic algorithms to find potential cause-and-effect relationships and likely upstream regulators that have a direct or indirect relationship with the DEGs (49). The *P* value of the enrichment score was used to evaluate the significance of the overlap between observed and predicted gene sets. The activation *z* score was used to assess the match between observed and predicted patterns of activation (≥2) and inhibition (≤–2). The networks of upstream regulators or DEGs regulated by the same upstream regulator were generated in IPA software. Enriched gene ontology analysis (count > 5 and *P* < 0.05) was performed using DAVID (Database for Annotation, Visualization and Integrated Discovery) Bioinformatics Resources 6.8 (https://david.ncifcrf.gov). Raw data files were deposited in the National Center of Biotechnology Information’s Gene Expression Omnibus (GEO) database (GEO GSE154926, https://www.ncbi.nlm.nih.gov/geo).

### Single-cell RNA-Seq analysis.

Human PMBCs were treated with sham control, LPS (100 ng/mL), or HSP70 (1 μg/mL) for 20 hours. Then, adherent and nonadherent cell populations were processed separately to enrich monocytes. Cell capture, cDNA generation, preamplification, and library preparation were conducted using the Chromium Single Cell 3′v2 Reagent Kit (10× Genomics) following the manufacturer’s instructions. Libraries were sequenced on an Illumina NextSeq 500. The quality assessment summary of PBMC samples is shown in Supplemental Table 5.

The total count was normalized as CPM and then logarithmized. Individual sample libraries were combined and processed as a single library. PMBCs containing more than 200 and fewer than 4000 unique features were retained, and cells with a large percentage of their counts attributed to mitochondrial genes were filtered out. The identity of cell clusters was based on the expression of genes known to be highly expressed by specific cell types (Supplemental Figure 2A). Cell clustering was performed by the Leiden graph-clustering method (50) and displayed in uniform manifold approximation and projection (UMAP) format. For trajectory inference, coarse-grained connectivity structures of cell clusters were mapped out using partition-based graph abstraction with computed diffusion pseudotime (51, 52). DEGs between the *BMP6^+^* monocyte cluster and the *CD14^++^CD16^–^* monocyte clusters were identified using a logistic regression model.

### Search strategy and study selection.

A systematic literature search was performed in PubMed in February 2021. Inclusion criteria were proteomics studies on salivary glands, saliva or serum/plasma from patients with SS, and particular studies focusing on each DAMP in SS. Review articles, case reports/series, and animal studies were excluded. In addition, the Human Salivary Proteome Wiki (https://salivaryproteome.nidcr.nih.gov) was searched for proteomics data on patients with SS. The detailed search strategy is shown in Supplemental Table 2.

### Measurement of extracellular HSP70 levels.

Serum HSP70 levels in study participants were determined by SomaScan (SomaLogic). Acinar, ductal, and HSG cells were cultured in OptiMEM (Thermo Fisher Scientific) starting 24 hours after transfection. Culture medium was collected and cleared by centrifugation at 2000*g* at 4°C for 10 minutes. The HSP70 concentration in the supernatant was measured by ELISA according to the manufacturer’s instructions (Proteintech, KE00059).

### Quantitative real-time reverse transcription PCR.

Total RNA was extracted from THP1 cells using the RNeasy Mini Kit (QIAGEN), treated with Turbo DNase (Thermo Fisher Scientific), and reverse-transcribed into cDNA using the High-Capacity RNA-to-cDNA Kit (Thermo Fisher Scientific). TaqMan Gene Expression Assays for *BMP6* (Hs01099594_m1), *TNFA* (Hs00174128_m1), and *ACTB* (Hs01060665_g1) were used to calculate transcript expression levels. Gene expression relative to *ACTB* was calculated using the ΔΔCt method. PCR cycles were performed on the Quantstudio3 Real-Time PCR System (Life Technologies, Thermo Fisher Scientific) under the following conditions: 2 minutes at 50°C, 10 minutes at 95°C, 50 cycles of 15 seconds at 95°C, and 1 minute at 60°C.

### Determination of BMP6 concentration.

Cultured THP1 cells were resuspended in PBS, lysed in 3 freeze-thaw cycles, and cleared by centrifugation at 17,000*g* at 4°C for 15 minutes. Culture medium was cleared by centrifugation at 2000*g* at 4°C for 10 minutes. The BMP6 concentration in the supernatant was determined by ELISA according to the manufacturer’s instructions (LifeSpan BioSciences, LS-F4538). The BMP6 concentration (μg/mL) was normalized to the total protein concentration (mg/mL) as measured by Bradford assay (Thermo Fisher Scientific).

### Western blotting.

Cultured acinar, ductal, and HSG cells were lysed in RIPA buffer with protease and phosphatase inhibitors (Thermo Fisher Scientific) and then cleared by centrifugation at 17,000*g* at 4°C for 15 minutes. Supernatants were heated at 97°C in NuPAGE LDS sample buffer for 10 minutes, resolved by SDS-PAGE, and electrophoretically transferred onto PVDF membranes (Thermo Fisher Scientific). Membranes were blocked with 5% nonfat dried milk at 25°C for 60 minutes and then incubated at 4°C overnight with one of the following primary antibodies: rabbit anti-LAMP3 antibody (Proteintech, 12632-1-AP); rabbit anti-HSP70 antibody (Cell Signaling Technology, 4872); or mouse anti–β-actin antibody (MilliporeSigma, A3854). After washing 3 times, the membranes were incubated with rabbit or mouse IgG HRP-linked whole antibody (MilliporeSigma) at 25°C for 1 hour. Signals were visualized using the Super Signal West Pico Chemiluminescent Substrate (Thermo Fisher Scientific).

### Isolation of EVs.

EVs were isolated according to the protocol described previously (26). Briefly, HSG cells were cultured in DMEM supplemented with 10% exosome-depleted FBS (Thermo Fisher Scientific) for 96 hours. Culture medium was collected and cleared by centrifugation at 2000*g* at 4°C for 30 minutes. Supernatant was incubated with Total Exosome Isolation Reagent (Thermo Fisher Scientific) at 4°C overnight. The mixture was centrifuged at 10,000*g* at 4°C for 60 minutes. Pellets, which contained the EVs, were resuspended in Opti-MEM (Thermo Fisher Scientific).

### Flow cytometry.

HSG cells were stained with phycoerythrin-conjugated mouse anti-LAMP1 antibody (BioLegend, 328607) on ice for 1 hour and then assessed with the BD Accuri (BD Biosciences). The intracellular Ca^2+^ concentration was determined using Fluo-4 calcium reagent (Thermo Fisher Scientific).

### Statistics.

Quantitative variables in in vitro and in vivo experiments were compared using a 2-tailed Student’s *t* test. When appropriate, multiple testing was corrected by Tukey’s or Dunnett’s method. Clinical data were assessed using the Wilcoxon test and Spearman’s correlation coefficient. *P* values of less than 0.05 were considered statistically significant. All analyses were performed using GraphPad Prism 8.0 (GraphPad Software).

### Study approval.

Individuals in the studies provided informed consent prior to the initiation of any study procedure. All clinical investigations were conducted in accordance with the Declaration of Helsinki principles. All studies using human samples were approved by the NIH’s IRB (ClinicalTrials.gov identifiers: NCT00001390, NCT02327884, and NCT00001196), the Brazilian Committee of Ethics in Research (37688914.2.0000.5440) and the Ethics Committee of the Hokkaido University Hospital (approval no. 014-0466) respective of the samples. All procedures involving live animals were approved on the basis of compliance with institutional guidelines and standard operating procedures (approval no. 18-863) following the NIH’s *Guide for the Care and Use of Laboratory Animals* (National Academies Press, 2011).

## Author contributions

YQM, HN, TT, TO, CQN, MN, TA, BMW, and JAC participated in the conception and design of the experiments. YQM, HN, TT, TO, PP, YJ, BNF, TJFP, DGM, HY, SSC, MK, SAA, CZ, FRO, ACFM, ARS, and EMR performed the experiments and data analyses. YQM, HN, TT, and JAC wrote the manuscript draft, which was revised and approved by all authors.

## Supplementary Material

Supplemental data

## Version 1 02/03/2022

In-Press Preview

## Version 2 03/15/2022

Electronic publication
