# Supplementary material for: Lysosomal exocytosis of HSP70 stimulates monocytic BMP6 expression in Sjögren’s syndrome
Source: J Clin Invest. 2022 Mar 15;132(6):e152780. doi: 10.1172/JCI152780 (PMC8920330; doi:10.1172/JCI152780)
Supplement: Supplemental data [file jci-132-152780-s163.pdf]

Supplemental Table 1. Differentially expressed genes in salivary glands between Sjögren's syndrome patients with high *BMP6* expression versus those with normal *BMP6* expression.

Gene symbol	Count per million	Log_2 fold change	*p* value	FDR
IGLVI-63	2.479239684	5.446	8.41E-06	0.028050458
UBD	3.416763525	5.205	4.89E-08	0.001686146
KRT4	2.809991105	5.191	1.08E-05	0.028050458
LOC105375724	3.809394049	3.578	2.19E-05	0.030829178
**LTA**	**2.001291919**	**3.365**	**0.00013041**	**0.049406587**
IGLV3-22	2.511103253	3.266	2.51E-05	0.031705253
**CXCL13**	**3.596567595**	**3.138**	**1.56E-05**	**0.028050458**
VPREB3	1.922862637	2.799	1.24E-05	0.028050458
**CXCL9**	**3.242140672**	**2.78**	**8.08E-06**	**0.028050458**
TCL1A	2.280466157	2.773	2.21E-05	0.030829178
TMEM163	1.919053457	2.675	9.61E-05	0.046670237
IGKV7-3	2.863533857	2.67	2.95E-05	0.033461111
**NCR3**	**1.758239343**	**2.658**	**4.63E-05**	**0.036352544**
**CCL19**	**4.877741778**	**2.613**	**9.43E-07**	**0.016252662**
IGKV1D-37	1.230044563	2.612	7.06E-05	0.041981695
**LTA**	**1.621659027**	**2.443**	**0.000128637**	**0.049276675**
**MMP9**	**2.405295351**	**2.361**	**8.68E-05**	**0.045060433**
CD52	5.74789247	2.356	2.01E-05	0.030829178
CD19	2.36792619	2.281	0.000107506	0.04691603
**PLA2G2D**	**3.920237621**	**2.169**	**0.000122961**	**0.049276675**
RP11-330H6.5	1.766939444	2.163	9.55E-05	0.046670237
PPP1R18	1.617340638	2.154	4.51E-05	0.036352544
IGKV1-8	4.652770241	2.144	8.64E-05	0.045060433
LTB	2.231687832	2.072	4.48E-05	0.036352544

Gene symbol	Count per million	Log_2 fold change	*p* value	FDR
IGKV2-10	1.707040236	2.034	1.43E-05	0.028050458
NKG7	4.076117313	2.013	7.70E-06	0.028050458
TIFAB	1.805860479	1.998	0.000104882	0.04691603
WDFY4	1.757147594	1.988	0.00010526	0.04691603
HCST	3.886703663	1.984	3.01E-05	0.033461111
**BCL2A1**	**2.379617337**	**1.981**	**5.10E-05**	**0.03700639**
CLECL1	2.216661299	1.972	0.00011509	0.047986684
ADAM19	3.888868174	1.943	3.49E-05	0.034783959
RBP5	2.090748562	1.924	1.17E-05	0.028050458
**PTGDS**	**5.927266531**	**1.893**	**3.55E-05**	**0.034783959**
IGKV1OR22-1	2.410195285	1.887	6.05E-05	0.039337364
HLA-DRB1	6.657364793	1.872	7.05E-06	0.028050458
**CMPK2**	**2.47488667**	**1.853**	**4.03E-06**	**0.028050458**
TMC8	2.052861999	1.851	9.45E-06	0.028050458
**POU2F2**	**3.306801345**	**1.834**	**5.15E-05**	**0.03700639**
GZMK	3.312458993	1.834	7.04E-05	0.041981695
**TFEC**	**2.385957817**	**1.822**	**9.75E-05**	**0.046670237**
LGALS2	3.304511646	1.797	1.48E-05	0.028050458
IGHV4-4	1.495989033	1.796	5.78E-05	0.038847864
**GZMA**	**2.88278428**	**1.78**	**1.48E-05**	**0.028050458**
CD3D	4.540737092	1.757	2.24E-05	0.030829178
**SLC15A3**	**1.95706469**	**1.729**	**0.000102935**	**0.04691603**
**ITGAX**	**4.663820874**	**1.723**	**2.67E-05**	**0.031705253**
SPIB	3.503693733	1.714	0.000112996	0.047986684
**OASL**	**3.118165737**	**1.704**	**8.65E-05**	**0.045060433**
P2RX5	1.696457884	1.675	0.000115527	0.047986684

Gene symbol	Count per million	Log_2 fold change	*p* value	FDR
CHST11	1.79255394	1.668	8.00E-05	0.045060433
RASGRP1	1.850078463	1.65	9.08E-05	0.045993801
CD72	2.128759224	1.644	2.53E-05	0.031705253
HLA-DRB3	3.192818499	1.639	6.53E-05	0.04096311
**ITGAL**	**3.416188199**	**1.628**	**3.37E-05**	**0.034783959**
ARHGAP9	2.758677331	1.609	3.74E-05	0.034836115
**CD69**	**1.943597729**	**1.598**	**1.12E-05**	**0.028050458**
TBC1D10C	2.316705466	1.57	5.86E-05	0.038847864
**CSF2RB**	**3.037698016**	**1.568**	**1.63E-05**	**0.028050458**
BMP6	1.62140251	1.518	0.000125796	0.049276675
LIMD2	2.747846772	1.515	6.68E-05	0.041103297
ANXA2R	2.705991112	1.486	8.57E-05	0.045060433
**CD8A**	**2.874337505**	**1.48**	**3.98E-05**	**0.036088675**
GPR65	2.336738777	1.464	4.42E-05	0.036352544
FCRL5	3.432757457	1.438	9.21E-05	0.045993801
CD37	4.247881191	1.433	6.70E-06	0.028050458
**TRAF1**	**2.193123799**	**1.432**	**0.000127498**	**0.049276675**
GVINP1	2.151973077	1.388	7.54E-05	0.04404202
**PTPN7**	**2.729516376**	**1.379**	**5.43E-05**	**0.037423639**
**CD48**	**3.875190034**	**1.379**	**0.000107124**	**0.04691603**
KIF20B	2.307512186	1.34	8.15E-05	0.045060433
SNX20	3.170497354	1.322	0.000101553	0.04691603
**SERPINB9**	**5.728634271**	**1.321**	**1.50E-05**	**0.028050458**
CERKL	2.198168143	1.312	4.79E-05	0.036686171
LY86	2.834416189	1.303	0.000127154	0.049276675
EPSTI1	3.513596227	1.298	8.23E-05	0.045060433

Gene symbol	Count per million	Log_2 fold change	*p* value	FDR
PYHIN1	2.216694521	1.292	0.000124394	0.049276675
**IL32**	**5.263001527**	**1.266**	**8.76E-05**	**0.045060433**
SFMBT2	2.048830941	1.246	4.57E-05	0.036352544
PCED1B	2.876793685	1.234	6.37E-05	0.040669271
BCL2	2.579611406	1.205	0.000102361	0.04691603
APOBEC3D	4.706302587	1.181	1.81E-05	0.029672261
SEMA4D	4.13171056	1.174	4.64E-05	0.036352544
GRK6	2.368965859	1.143	2.62E-05	0.031705253
GLCCI1	3.598950935	1.106	5.12E-05	0.03700639
**IRF7**	**2.585094745**	**1.08**	**5.26E-05**	**0.03700639**
MEX3C	3.180689227	0.932	0.000111219	0.047929645
KRT38	1.578279786	-3.886	0.000123737	0.049276675
RPL3P4	3.677699188	-4.622	3.63E-05	0.034783959
HNRNPA1P40	4.294464998	-5.5	8.46E-06	0.028050458
BPIFA1	3.613842413	-5.681	3.20E-05	0.034458527

Genes shown in bold were reported to be regulated by LPS.

Supplemental Table 2. Literature searching strategy and result.

	Search term	Hit	Match
"Sjogren syndrome"	+ "**Proteomics**"	183	**17**
"Sjogren syndrome"	+ "**HMGB1**"	10	**3**
	+ "**Heat shock protein**"	60	**2**
	+ "**Hyaluronan**"	36	**1**
	+ "**Fibronectin**"	30	**1**
	+ "Histone"	88	0
	+ "Fibrinogen"	25	0
	+ "S100"	9	0
	+ "Defensin"	9	0
	+ "Syndecans"	7	0
	+ "Tenascin C"	4	0
	+ "Decorin"	2	0
	+ "Heparan sulfate"	2	0
	+ "Biglycan"	1	0
	+ "Granulysin"	0	0
	+ "Glypicans"	0	0
	+ "HMGN1"	0	0

HMGB1, high mobility group box 1; HMGN1, high mobility group nucleosome binding domain 1.

Supplemental Table 3. Potential TLR4 ligands reported to be increased in Sjögren's syndrome (SS) patients.

Reference		Sample	Potential TLR4 ligands increased in SS
**Human Salivary Proteome Wiki ***		**Saliva**	**HSP70, S100, Histones**
**Sembler-Møller ML (1)**	**2020**	**Saliva**	**HMGB1**
**Wei P (2)**	**2020**	**Saliva**	
**Cecchettini A (3)**	**2019**	**Saliva**	**HSP70, S100, Histones**
**Aqrawi LA (4)**	**2019**	**Saliva**	
**Hall SC (5)**	**2017**	**Saliva**	**Histones**
**Aqrawi LA (6)**	**2017**	**Saliva**	**HSP70**
**Deutsch O (7)**	**2015**	**Saliva**	**HSP70, S100, Histones, Fibrinogen**
**Ambatipudi KS (8)**	**2012**	**Saliva**	**HSP70, S100, Histones, Fibrinogen**
**Baldini C (9)**	**2011**	**Saliva**	**S100**
**Fleissig Y (10)**	**2009**	**Saliva**	**S100, Fibrinogen**
Silvestre FJ (11)	2009	Saliva	Fibronectin
**Peluso G (12)**	**2007**	**Saliva**	**Defensin**
**Hu S (13)**	**2007**	**Saliva**	
**Giusti L (14)**	**2007**	**Saliva**	
**Ryu OH (15)**	**2006**	**Saliva**	
Tishler M (16)	1998	Saliva	Hyaluronan
**Hjelmervik TO (17)**	**2009**	**MSG**	**HSP60, HSP70, HSP90, Decorin**
Ek M (18)	2006	MSG	HMGB1
**Bodewes ILA (19)**	**2019**	**Serum**	
Kanne AM (20)	2018	Serum	HMGB1
**Nishikawa A (21)**	**2016**	**Serum**	
Bårdsen K (22)	2016	Plasma	HSP90
Dupire G (23)	2012	Serum	HMGB1
Aragona P (24)	1999	Serum	HSP60

Proteomic studies are indicated as bold. HSP, heat shock protein; HMGB1, high mobility group box 1; MSG, minor salivary gland. *https://salivaryproteome.nidcr.nih.gov.

Supplemental Table 4. Demographic and clinical characteristics between Sjögren's syndrome patients with high *BMP6* expression (n = 20) versus those with normal *BMP6* expression (n = 23).

Characteristics	High *BMP6*	Normal *BMP6*	p value*
Age, years	55 ± 12	50 ± 16	0.230
Female, *n (%)*	20 (100)	23 (100)	-
Low salivary flow (<0.1 mL/min), *n (%)*	14 (70)	16 (70)	0.975
Positive Schirmer's test (≤5mm/5min), *n (%)*	10 (50)	9 (39)	0.474
Anti-SSA Ab positive, *n (%)*	14 (70)	16 (70)	0.975
Anti-SSB Ab positive, *n (%)*	11 (55)	11 (48)	0.639
Focus score	1.2 ± 1.3	1.6 ± 1.1	0.264
Prior medications during 3 months before recruitment			
Glucocorticoids, *n (%)*	9 (45)	7 (30)	0.361
Immunosuppressants†, *n (%)*	9 (45)	6 (26)	0.219
Hydroxychloroquine, *n (%)*	3 (15)	1 (4)	0.323

†Immunosuppressants include methotrexate, azathioprine, cyclosporine and leflunomide.

*Fisher's exact test for categorical variables and *t*-test for continuous variables.

Supplemental Table 5. Quality assessment summary of PBMCs samples.

	Adherent			Non-adherent		
	Sham	LPS	HSP70	Sham	LPS	HSP70
Number of cells	9272	11844	32415	12005	9547	11674
Mean reads per cell	8011	25671	32590	10577	15112	37946
Median reads per cell	901	1715	1506	1052	1315	1424
Number of reads	74×10^6	304×10^6	1056×10^6	127×10^6	144×10^6	443×10^6
Valid Barcodes	96.5%	97.0%	96.6%	97.0%	96.9%	96.1%
Valid UMIs	99.9%	99.9%	99.9%	99.9%	99.9%	99.9%
Sequencing Saturation	32.0%	53.1%	59.2%	44.9%	50.3%	74.7%
Reads Mapped to Genome	97.6%	97.6%	97.5%	97.3%	97.2%	97.4%
Reads Mapped to Transcriptome	58.5%	60.8%	57.8%	56.6%	55.5%	56.7%
Fraction Reads in Cells	94.7%	94.7%	86.0%	93.2%	93.0%	94.5%
Total Genes Detected	19619	21756	23764	20530	20476	21875
Median UMI Counts per Cell	2048	4949	4782	2537	3293	4188

UMI, unique molecular identifier.

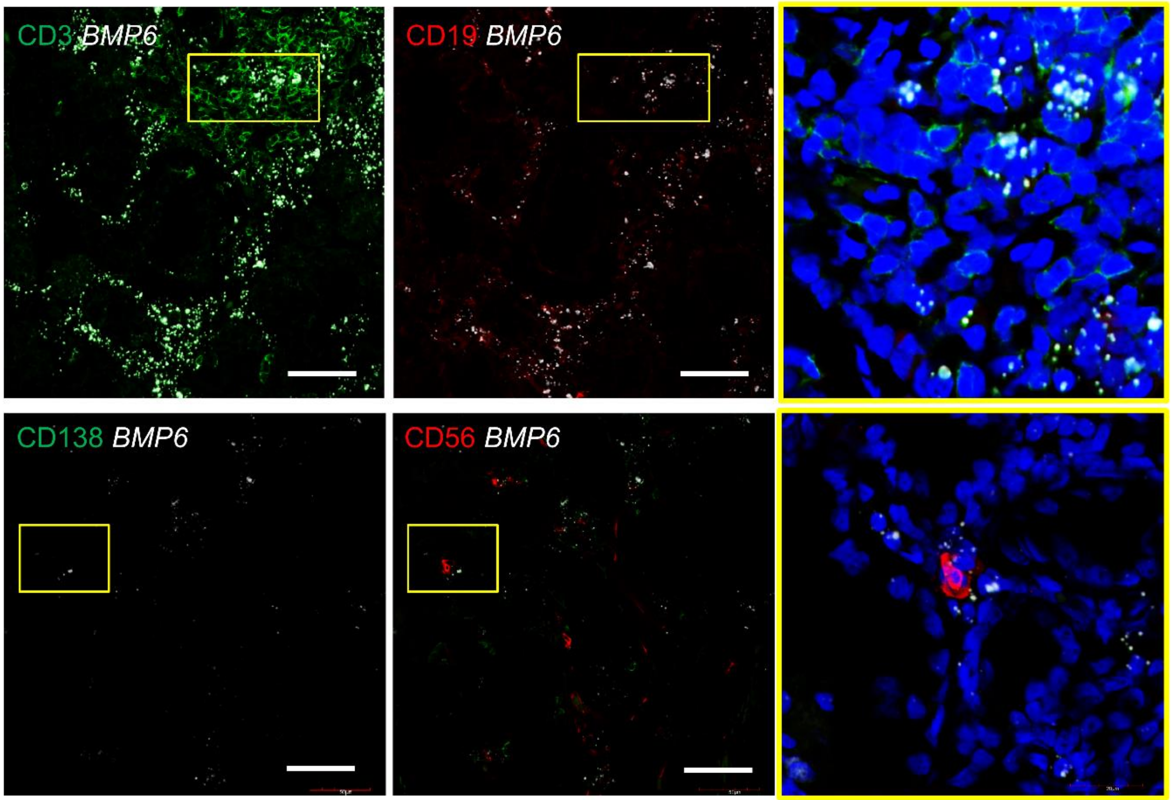

Supplemental Figure 1. *BMP6* mRNA expressing cells in salivary glands of patients with Sjögren's syndrome. Representative images of dual *in situ* hybridization for *BMP6* (white) and immunofluorescence for CD3 (green), CD19 (red), CD138 (green) or CD56 (red) on labial minor salivary gland sections. Scale bars = 50 µm.

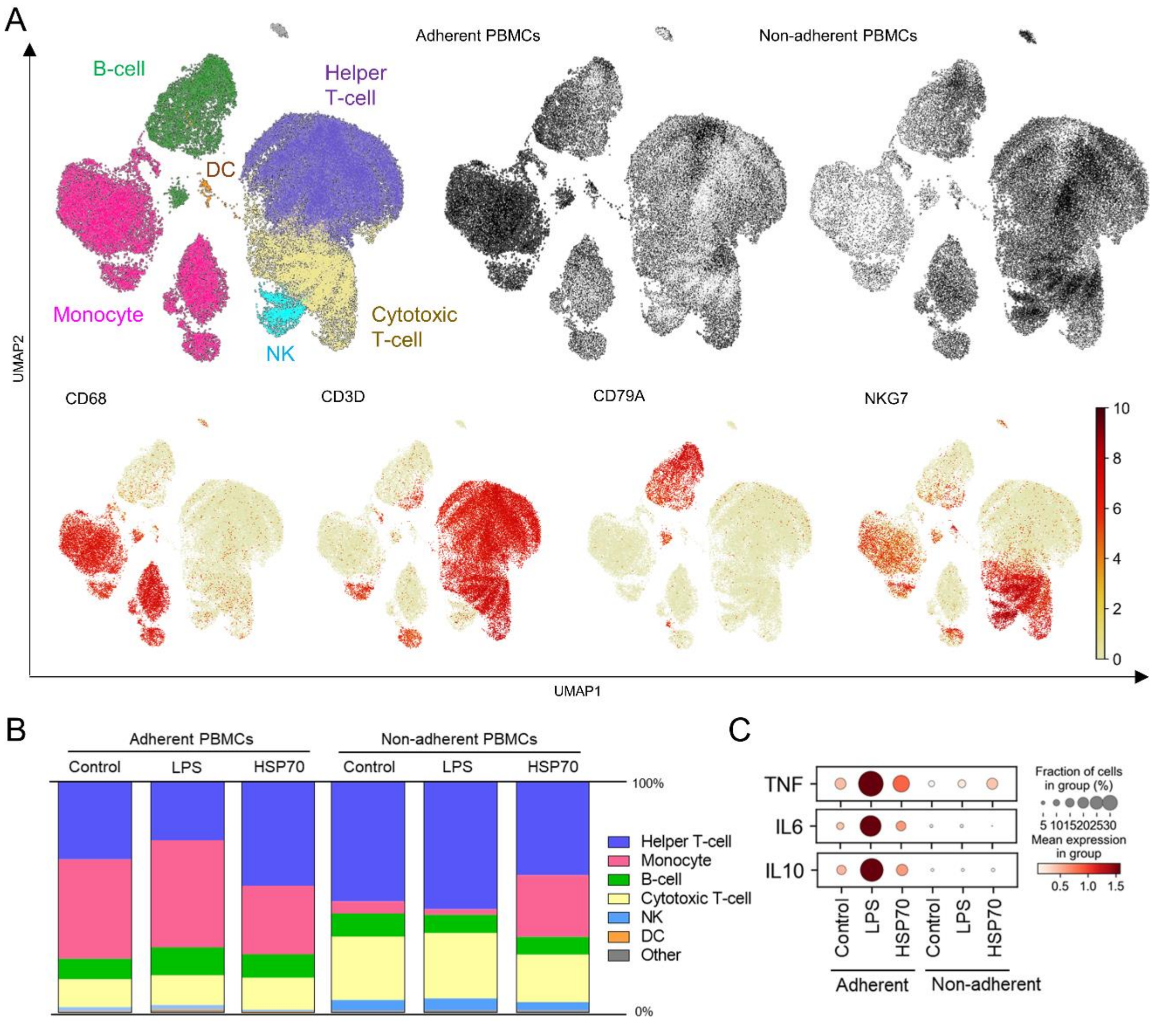

Supplemental Figure 2. Single-cell RNA sequencing libraries of human PBMCs. PBMCs were captured 20 hours after treatment with sham control, LPS or HSP70. The adherent and non-adherent populations were processed separately. (**A**) Annotated PBMCs are displayed in Uniform Manifold Approximation and Projection (UMAP) format with distribution of cells expressing the representative cell markers. (**B**) Proportion of each cell type among adherent and non-adherent groups. (**C**) Proportion and relative expression of indicted genes among adherent and non-adherent groups.

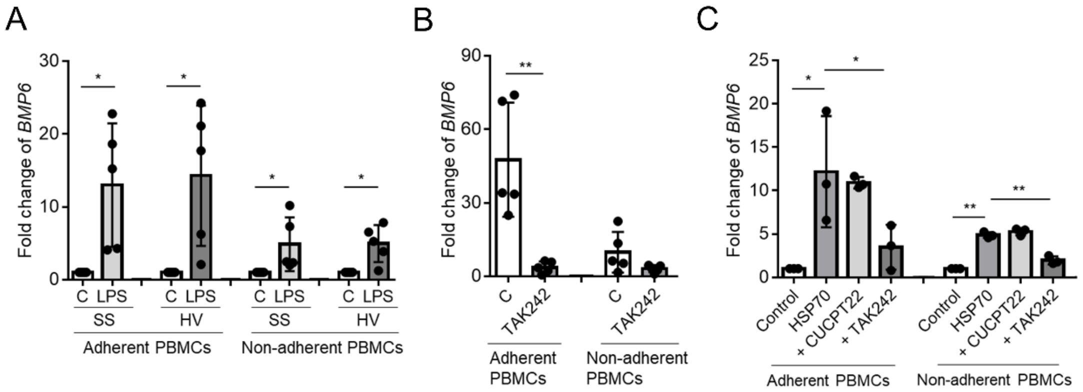

Supplemental Figure 3. ***BMP6*** **expression is upregulated via TLR4 pathway in human PBMCs.** (**A**) Human PBMCs isolated from patients with Sjögren's syndrome (SS, $n = 5$) or healthy volunteers (HVs, $n = 5$) were treated with LPS (100 ng/mL) for 20 hours. (**B**) PBMCs from HVs ($n = 5$) were treated with LPS ± TAK242 (40 μM) for 20 hours. (**C**) PBMCs from HVs ($n = 3$) were treated with recombinant HSP70 (1 μg/mL) ± CUCPT22 (20 μM) or TAK242 (40 μM) for 20 hours. *BMP6* transcript was quantified using $\Delta\Delta$Ct method relative to *ACTB* in adherent and non-adherent population. Values shown are mean ± SD. $*p < 0.05$, $**p < 0.01$, $***p < 0.001$, *t*-test with multiple testing correction using Dunnett's method.

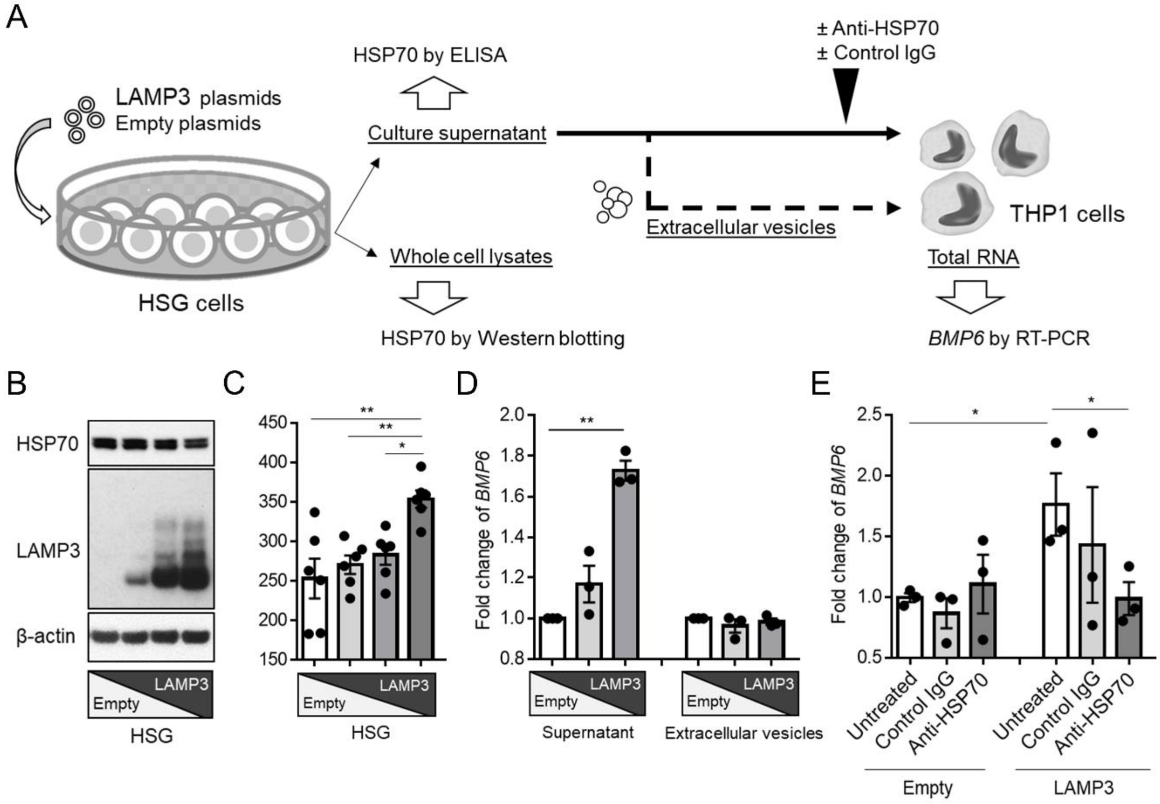

Supplemental Figure 4. *BMP6* expression is not stimulated by extracellular vesicles from LAMP3-overexpressing HSG cells (**A**) Schematic methods of the following *in vitro* assays. (**B**) Representative Western blot with indicated antibodies using lysate of HSG cells 72 hours after transfection with empty and/or *LAMP3* expression plasmids. (**C**) HSP70 concentration in culture supernatant collected 96 hours after transfection. (**D**) Culture supernatant of HSG cells was collected 96 hours after transfection with empty and/or *LAMP3* expression plasmids and extracellular vesicles were isolated from supernatant. THP1 cells were treated with unfractionated supernatant or isolated EVs for 20 hours. (**E**) THP1 cells were treated with culture supernatant of HSG cells ± HSP70 neutralizing antibody (1 µg/mL) or control IgG (1 µg/mL). *BMP6* transcript was quantified using $\Delta\Delta$Ct method relative to *ACTB.* Values shown are mean ± SEM from three or five independent experiments. *$p < 0.05$, **$p < 0.01$, *t*-test with multiple testing correction using Tukey's method.

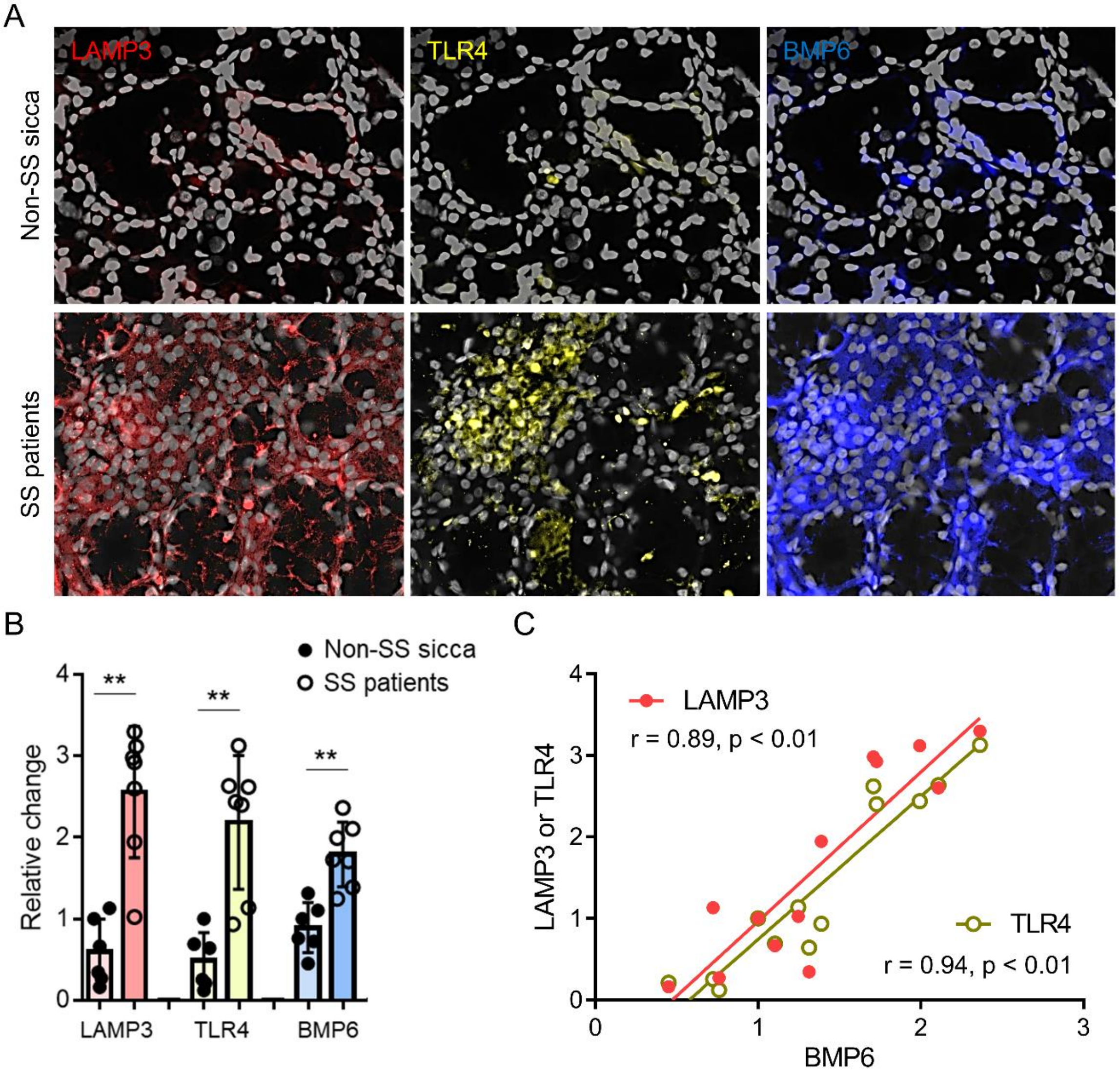

Supplemental Figure 5. LAMP3, TLR4 and BMP6 expression in salivary glands. (**A**) Representative images of immunofluorescence for LAMP3 (red, left panel), TLR4 (yellow, center panel) or BMP6 (blue, right panel) and nucleus (DAPI, gray) on labial minor salivary gland sections from patients with Sjögren's syndrome (SS, $n = 7$) or non-SS sicca. ($n = 6$). Original magnification: 40x. (**B**) Bar chart showing mean (± SD) relative change of each protein expression area. **$p < 0.01$, *t*-test. (**C**) Correlation between BMP6 and LAMP3 or TLR4 expression in labial minor salivary glands.

REFERENCES

1. Sembler-Møller ML, Belstrøm D, Locht H, Pedersen AML. Proteomics of saliva, plasma, and salivary gland tissue in Sjögren's syndrome and non-Sjögren patients identify novel biomarker candidates. *J Proteomics* 2020;225:103877.
2. Wei P, Xing Y, Li B, Chen F, Hua H. Proteomics-based analysis indicating α-enolase as a potential biomarker in primary Sjögren's syndrome. *Gland Surg* 2020;9:2054-63.
3. Cecchettini A, Finamore F, Ucciferri N, Donati V, Mattii L, Polizzi E, et al. Phenotyping multiple subsets in Sjögren's syndrome: a salivary proteomic SWATH-MS approach towards precision medicine. *Clin Proteomics* 2019;16:26.
4. Aqrawi LA, Galtung HK. Proteomic and histopathological characterisation of sicca subjects and primary Sjögren's syndrome patients reveals promising tear, saliva and extracellular vesicle disease biomarkers. *Arthritis Res Ther* 2019;21:181.
5. Hall SC, Hassis ME, Williams KE, Albertolle ME, Prakobphol A, Dykstra AB, et al. Alterations in the Salivary Proteome and N-Glycome of Sjögren's Syndrome Patients. *J Proteome Res* 2017;16:1693-705.
6. Aqrawi LA, Galtung HK, Vestad B, Øvstebø R, Thiede B, Rusthen S, et al. Identification of potential saliva and tear biomarkers in primary Sjögren's syndrome, utilising the extraction of extracellular vesicles and proteomics analysis. *Arthritis Res Ther* 2017;19:14.
7. Deutsch O, Krief G, Konttinen YT, Zaks B, Wong DT, Aframian DJ, et al. Identification of Sjögren's syndrome oral fluid biomarker candidates following high-abundance protein depletion. *Rheumatology (Oxford)* 2015;54:884-90.
8. Ambatipudi KS, Swatkoski S, Moresco JJ, Tu PG, Coca A, Anolik JH, et al. Quantitative proteomics of parotid saliva in primary Sjögren's syndrome. *Proteomics* 2012;12:3113-20.
9. Baldini C, Giusti L, Ciregia F, Da Valle Y, Giacomelli C, Donadio E, et al. Proteomic analysis of saliva: a unique tool to distinguish primary Sjögren's syndrome from secondary Sjögren's syndrome and other sicca syndromes. *Arthritis Res Ther* 2011;13:R194.

10. Fleissig Y, Deutsch O, Reichenberg E, Redlich M, Zaks B, Palmon A, et al. Different proteomic protein patterns in saliva of Sjögren's syndrome patients. *Oral Dis* 2009;15:61-8.
11. Silvestre FJ, Puente A, Bagán JV, Castell JV. Presence of fibronectin peptides in saliva of patients with Sjögren's syndrome: a potential indicator of salivary gland destruction. *Med Oral Patol Oral Cir Bucal* 2009;14:e365-70.
12. Peluso G, De Santis M, Inzitari R, Fanali C, Cabras T, Messana I, et al. Proteomic study of salivary peptides and proteins in patients with Sjögren's syndrome before and after pilocarpine treatment. *Arthritis Rheum* 2007;56:2216-22.
13. Hu S, Wang J, Meijer J, Ieong S, Xie Y, Yu T, et al. Salivary proteomic and genomic biomarkers for primary Sjögren's syndrome. *Arthritis Rheum* 2007;56:3588-600.
14. Giusti L, Baldini C, Bazzichi L, Ciregia F, Tonazzini I, Mascia G, et al. Proteome analysis of whole saliva: a new tool for rheumatic diseases--the example of Sjögren's syndrome. *Proteomics* 2007;7:1634-43.
15. Ryu OH, Atkinson JC, Hoehn GT, Illei GG, Hart TC. Identification of parotid salivary biomarkers in Sjogren's syndrome by surface-enhanced laser desorption/ionization time-of-flight mass spectrometry and two-dimensional difference gel electrophoresis. *Rheumatology (Oxford)* 2006;45:1077-86.
16. Tishler M, Yaron I, Shirazi I, Yaron M. Salivary and serum hyaluronic acid concentrations in patients with Sjögren's syndrome. *Ann Rheum Dis* 1998;57:506-8.
17. Hjelmervik TO, Jonsson R, Bolstad AI. The minor salivary gland proteome in Sjögren's syndrome. *Oral Dis* 2009;15:342-53.
18. Ek M, Popovic K, Harris HE, Naucler CS, Wahren-Herlenius M. Increased extracellular levels of the novel proinflammatory cytokine high mobility group box chromosomal protein 1 in minor salivary glands of patients with Sjogren's syndrome. *Arthritis Rheum* 2006;54:2289-94.
19. Bodewes ILA, van der Spek PJ, Leon LG, Wijkhuijs AJM, van Helden-Meeuwsen CG, Tas L, et al. Fatigue in Sjögren's Syndrome: A Search for Biomarkers and Treatment Targets. *Front Immunol* 2019;10:312.
20. Kanne AM, Julich M, Mahmutovic A, Troster I, Sehnert B, Urbonaviciute V, et al. Association of High Mobility Group Box Chromosomal Protein 1 and Receptor for

Advanced Glycation End Products Serum Concentrations With Extraglandular Involvement and Disease Activity in Sjogren's Syndrome. *Arthritis Care Res (Hoboken)* 2018;70:944-8.

21. Nishikawa A, Suzuki K, Kassai Y, Gotou Y, Takiguchi M, Miyazaki T, et al. Identification of definitive serum biomarkers associated with disease activity in primary Sjögren's syndrome. *Arthritis Res Ther* 2016;18:106.
22. Bårdsen K, Nilsen MM, Kvaløy JT, Norheim KB, Jonsson G, Omdal R. Heat shock proteins and chronic fatigue in primary Sjögren's syndrome. *Innate Immun* 2016;22:162-7.
23. Dupire G, Nicaise C, Gangji V, Soyfoo MS. Increased serum levels of high-mobility group box 1 (HMGB1) in primary Sjögren's syndrome. *Scand J Rheumatol* 2012;41:120-3.
24. Aragona P, Magazzù G, Macchia G, Bartolone S, Di Pasquale G, Vitali C, et al. Presence of antibodies against Helicobacter pylori and its heat-shock protein 60 in the serum of patients with Sjögren's syndrome. *J Rheumatol* 1999;26:1306-11.
